# Bisecting *N*-Acetylglucosamine of the *N*-Glycan of Immunoglobulin G Does Not Affect Binding to Fc Gamma Receptors

**DOI:** 10.1021/acschembio.4c00807

**Published:** 2025-02-19

**Authors:** Gerlof P. Bosman, Inèz D. Stoof, Hans P. Bastiaansen, Linda Quarles van Ufford, Justyna M. Dobruchowska, Jan-Willem H. Langenbach, Bhargavi M. Boruah, Kelley W. Moremen, Arthur E. H. Bentlage, Suzanne N. Lissenberg-Thunnissen, Gestur Vidarsson, Geert-Jan Boons

**Affiliations:** † Chemical Biology and Drug Discovery, Utrecht Institute for Pharmaceutical Sciences, Utrecht University, 3584 CG Utrecht, The Netherlands; ‡ Complex Carbohydrate Research Center, University of Georgia, 315 Riverbend Road, Athens, Georgia 30602, United States; § Department of Biochemistry and Molecular Biology, The University of Georgia, Athens, Georgia 30602, United States; ∥ Immunoglobulin Research Laboratory, Sanquin Research, 1066 CX Amsterdam, The Netherlands; ⊥ Department of Biomolecular Mass Spectrometry and Proteomics, Utrecht University, 3584 CG Utrecht, The Netherlands; # Bijvoet Center for Biomolecular Research, Utrecht University, 3584 CG Utrecht, The Netherlands; ∇ Department of Chemistry, The University of Georgia, Athens, Georgia 30602, United States

## Abstract

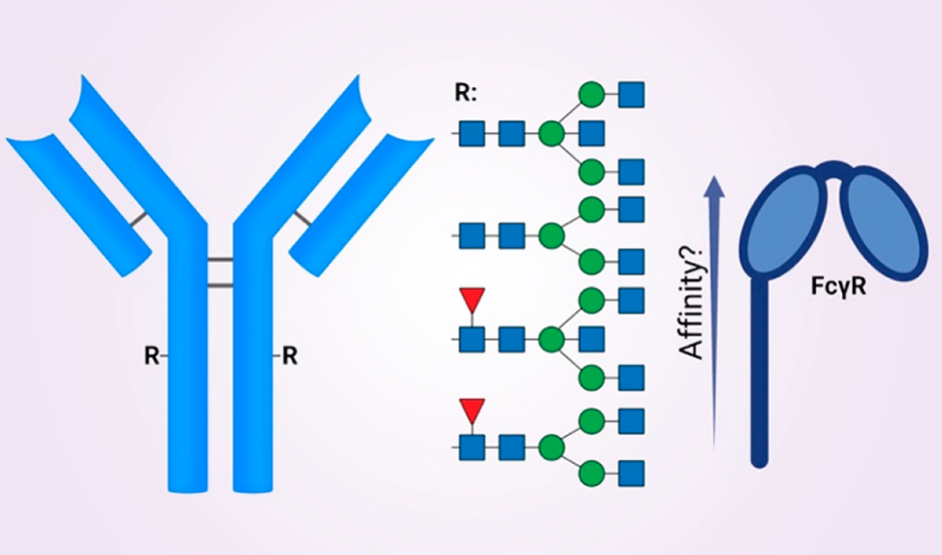

Monoclonal antibodies (mAb) produced in 1,4-mannosyl-glycoprotein 4-*N*-acetylglucosaminyltransferase (MGAT3) overexpressing cell lines have superior *in vitro* and *in vivo* activities. The *N*-glycan of the Fc-region of these mAbs have increased levels of bisecting *N*-acetylglucosamine (GlcNAc) and reduced core-fucosylation. Although a reduction in core-fucosylation will improve FcγRIIIa binding and antibody-dependent cellular cytotoxicity (ADCC) activity, the influence of bisecting GlcNAc on these activities has been difficult to probe. Here, we describe the preparation of a unique series of homogeneous glycoforms of trastuzumab (Herceptin) with and without core-fucose and with and without bisecting GlcNAc and examine binding to a comprehensive panel of Fcγ receptors. The glycoforms of trastuzumab were prepared by treatment with wild-type Endo-S2 to cleave the chitobiose core of the *N*-glycan to leave GlcNAc-Fuc that was exposed to an α-fucosidase to provide trastuzumab-GlcNAc. Glycan oxazolines with and without bisecting GlcNAc were prepared by enzymatic remodeling of a sialoglycopeptide isolated from egg yolk powder, which were employed in transglycosylations with trastuzumab-GlcNAc and trastuzumab-GlcNAc-Fuc catalyzed by Endo-S2 D184M resulting in well-defined glycoforms. As expected, core-fucosylation had a major effect on FcγRIIIa binding, which was not influenced by the presence of bisecting GlcNAc. It was found that an A2-glycan (GlcNAc_2_Man_3_GlcNAc_2_) modified by bisecting GlcNAc cannot be core-fucosylated by FUT8. Thus, bisecting GlcNAc has only an indirect influence on FcγRIIIa binding and subsequent ADCC activity by inhibiting core-fucosylation. The results described here provide an understanding of the properties of therapeutic monoclonal antibodies.

## Introduction

Monoclonal antibodies (mAbs) are important therapeutic modalities for the treatment of various diseases such as cancer, infections, and hematological and autoimmunity disorders. Immunoglobulin G (IgG) bears a single *N*-glycan at N297 which is mainly of the biantennary complex type that can vary in the degree of core-fucosylation, galactosylation, sialylation, and bisecting GlcNAc.^1−3^ The Fc-glycan is located at the interface of engaging effector ligands and can influence antibody-dependent cellular cytotoxicity (ADCC) initiated through IgG Fc-receptor (FcγR) binding and complement-dependent cytotoxicity (CDC) initiated through C1q binding. It is well established that core-fucosylation impacts FcγR binding, while galactosylation has an influence on C1q binding.^4,5^ Whether bisecting GlcNAc has a direct effect on FcγR binding has, however, not been fully elucidated.

Late-stage processing of *N*-glycans in the medial Golgi involves installation of a GlcNAc moiety at the 1,3-mannose arm of a Man5-glycan by the enzyme 1,3-mannosyl-glycoprotein 2-*N*-acetylglucosaminyltransferase (MGAT1) to give GlcNAcMan_5_GlcNAc_2_, which is followed by hydrolysis of the two mannosyl residues at the 1,6-mannose arm. Next, GlcNAc is added by 1,6-mannosyl-glycoprotein 2--*N*-acetylglucosaminyltransferase (MGAT2) to yield a biantennary precursor: the A2-glycan. The antennae of the A2-glycan can be further extended by galactosyl- and sialyl-transferases to give complex biantennary glycans. In the *trans* Golgi, core-fucose can be installed on GlcNAcMan_5_GlcNAc_2_ by 1,6-fucosyltransferase (FUT8). Furthermore, 1,4-mannosyl-glycoprotein 4--N-acetylglucosaminyltransferase (MGAT3) can add a bisecting GlcNAc moiety at the core β-mannoside of GlcNAcMan_5_GlcNAc_2_ or the A2-glycan, after which it can maturate into a complex glycan. Both FUT8 and MGAT3 act on similar precursors, which inevitably causes an interplay between the action of the two enzymes. It has, for example, been demonstrated that core-fucosylation is inhibited when bisected *N*-glycans are exposed to a Golgi-rich fraction of porcine liver extracts containing FUT8.^6^

NMR analyses of synthetic *N*-glycans with and without bisecting GlcNAc have revealed that a bisecting GlcNAc residue restricts the conformational space of both the 1,3- and 1,6-mannose antennae. Glycan flexibility is restricted due to the 1,6-branched Man acquiring a so-called “back-fold” conformation. Changes in conformational properties due to bisecting GlcNAc moiety can impact protein-glycan binding.^7^ The lectins Calsepa and PHA-E recognize the back-fold conformer, and crystal structures showed a back-folded glycan structure when bound to the lectins.^8^ This highlights the importance of glycan conformation for molecular recognition, and it is conceivable that the back-fold conformer may influence interactions with FcγRs.

It is well established that core-fucosylation of the *N*-glycan at N297 of IgG sterically hinders intermolecular carbohydrate-carbohydrate interactions with the N162 glycan of FcγRIIIa, thereby reducing the ADCC activity. For example, the anti-CD20 antibody ublituximab produced in the rat myeloma cell line, YB2/0 cell line, which has a 10-fold lower transcript level of FUT8 compared to the expression in wild-type CHO cells, has a 90% decrease in core-fucosylation. As a result, the mAb exhibits more than a 50-fold increase in ADCC activity.^9^ A reduction in core-fucosylation has also been achieved by coexpression of MGAT3. Generally, it results in an increase in the incorporation of bisecting GlcNAc to 50–70%^10−12^ and a reduction of core-fucosylation to just 10%.^10,13^ This approach has been used to produce imgatuzumab, lumretuzumab, and obinutuzumab. An antineuroblastoma IgG1 produced in a MGAT3 coexpressing cell line has significantly improved ADCC activity.^10,13,14^ Enhanced FcγRIIIa binding, likely driven by reduced core fucosylation due to increased bisecting GlcNAc, may contribute to improved ADCC. Furthermore, it has been shown that afucosylated trastuzumab (Herceptin) with or without a bisecting GlcNAc exhibits comparable affinities for FcγRIIIa and similar ADCC activity.^15^ Although these results indicate that core-fucosylation has a major impact on FcγR binding, a possible role of bisecting GlcNAc in modulating the binding of FcγRs is lacking.

Although glycoengineering of antibodies through coexpression of glycosyltransferases has provided an understanding of how glycan composition influences FcγR binding, this strategy generally gives heterogeneity in mAb glycosylation, hampering drawing unambiguous conclusions. An alternative strategy for the preparation of glycoforms of monoclonal antibodies is based on the treatment of a heterogeneous mixture of glycoforms by an endo--*N*-acetylglucosaminidase (ENGase) to leave a GlcNAc residue at *N*-linked glycosylation sites. In a second step, a glycan of choice can be attached to this GlcNAc moiety by employing a mutant ENGase and a well-defined glycan having its anomeric center modified as an oxazoline.^16−19^

**Figure 1:**
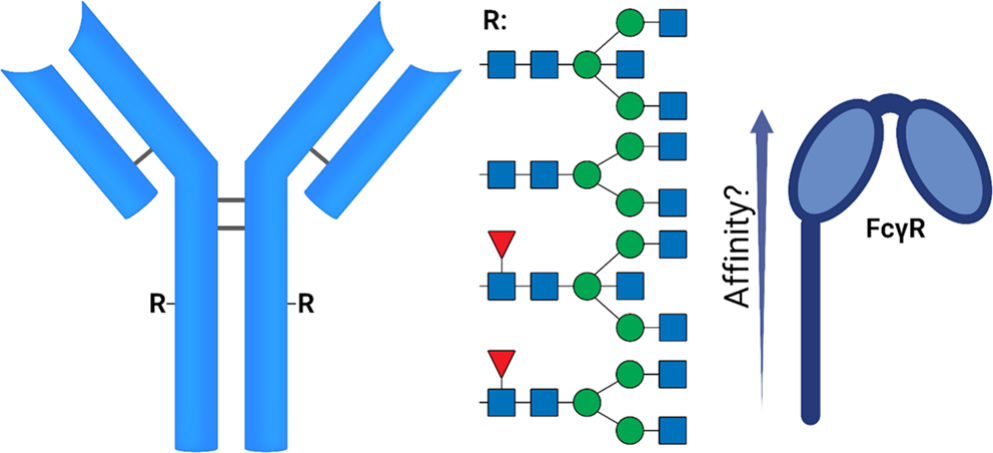
Four glycoforms of trastuzumab (Herceptin) with and without core-fucose and with and without bisecting GlcNAc are synthesized for assessing binding to FcγRs.

In the present study, we aimed to prepare a unique series of homogeneous glycoforms of trastuzumab (Herceptin) with and without core-fucose and with and without bisecting GlcNAc using ENGase-mediated transglycosylation (Figure 1). We envisaged that treatment of trastuzumab with wild-type Endo-S2 would cleave the chitobiose core, leaving a GlcNAc-Fuc moiety that upon further treatment with the α-fucosidase FucH^20^ would yield trastuzumab-GlcNAc. Transglycosylation of semisynthetic glycan oxazolines and the ENGase mutant, Endo-S2 D184M,^21^ would result in well-defined glycoforms, making it possible to investigate in a systematic manner whether FcγR binding is influenced solely by a lack of core-fucose or also affected by the presence of bisecting GlcNAc.

## Results and Discussion

### Preparation of Activated Glycan Oxazolines 5, 6 and mAbs 8, 9

**Figure 2:**
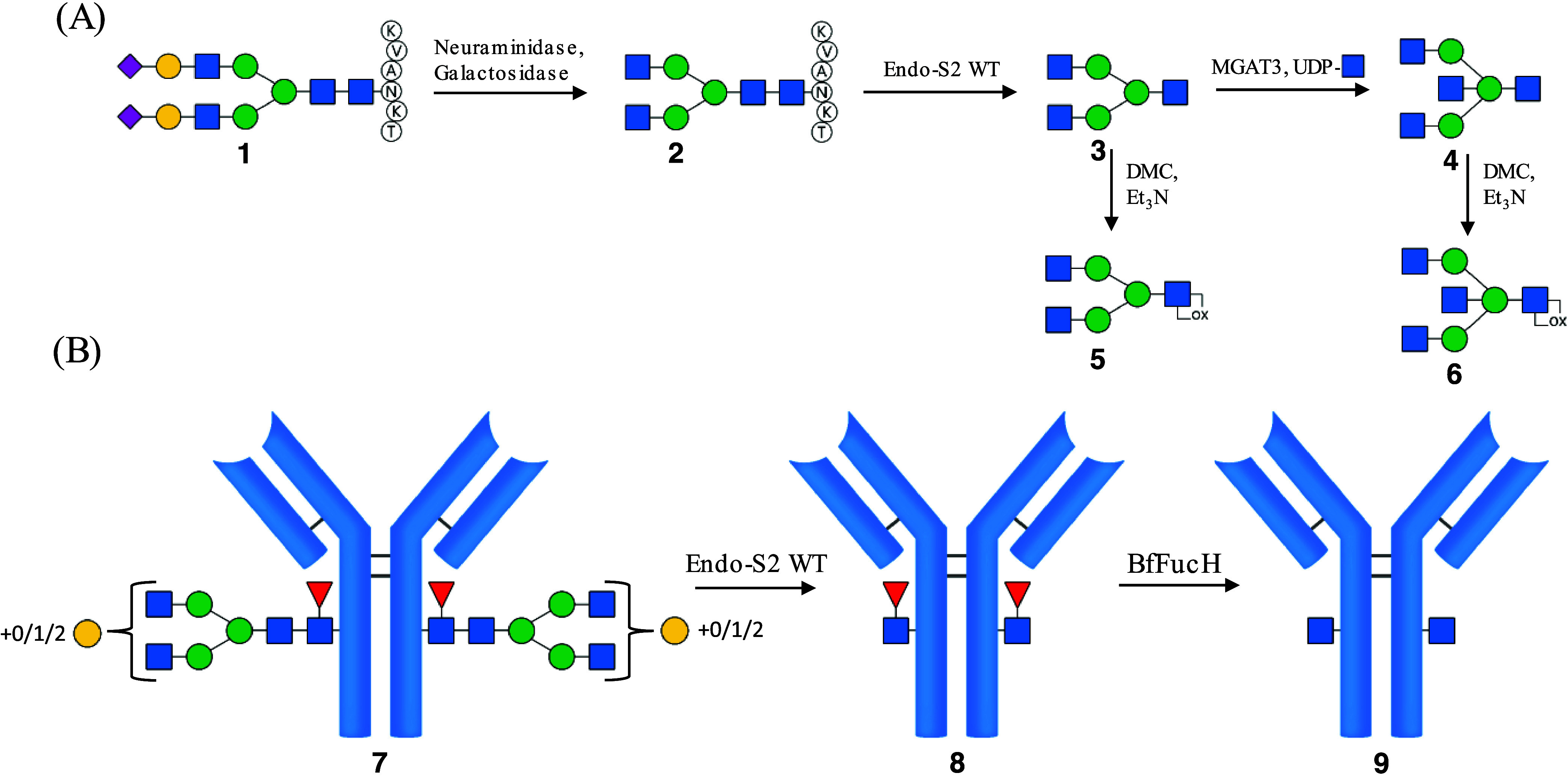
Preparation of glycan oxazolines **5** and **6** and mAbs **8** and **9**. (A) SGP is trimmed with neuraminidase (*Clostridium perfringens*) and galactosidase BgaA (*Streptococcus pneumoniae*) to obtain the A2-peptide **2**, which was treated with wild-type Endo-S2 yielding the A2-glycan **3**. The A2-glycan is activated with DMC and triethylamine (Et_3_N) to form the A2-oxazoline **5**. Alternatively, the A2-glycan can be modified by MGAT3 to A3B-glycan **4**, which is activated with DMC and Et_3_N to A3B-oxazoline **6**. (B) Wild-type trastuzumab (Herceptin) is treated with wild-type Endo-S2 to yield GlcNAc-Fuc mAb **8** and can further be modified to GlcNAc-Fuc mAb **9** by treating the mAb with BfFucH. NMR data of the glycans are found in the Experimental Section and Figures S1–S5, and MS spectra of the mAbs **7**–**9** are found in Figures S7–S9.

The preparation of well-defined glycoforms of trastuzumab variants **10**–**13** started by the synthesis of glycan oxazolines **5** and **6** and the trimming of the antibody to glycoforms **8** and **9**. For this purpose, the galactosidase BgaA, α-fucosidase BfFucH, and wild-type Endo-S2 and the glycosynthase Endo-S2 D184M were expressed in *Escherichia coli*,^20−22^ and MGAT3 was produced by transient transfection of HEK293F cells using reported approaches.^23^ To prepare oxazolines **5**(24) and **6**, we started with the extraction of a sialoglycopeptide SGP (**1**) from egg yolk powder,^25^ which was trimmed by a neuraminidase and galactosidase BgaA to obtain the A2-glycopeptide **2** (Figure 2A). The peptide was released using wild-type Endo-S2 to yield the A2-glycan **3**, which was converted into an oxazoline using 2-chloro-1,3-dimethylimidazolinium chloride (DMC)^26^ to provide A2-oxazoline **5**. Another portion of the A2-glycan was incubated with recombinant MGAT3 and UDP-GlcNAc to yield the A3B-glycan **4**, which was treated with DMC to provide A3B-oxazoline **6**. Glycans **3**–**6** were characterized by LC-MS, which provided the expected masses, and the structures were further confirmed by NMR (Figures S2–S5). The ^1^H and ^13^C chemical shifts were assigned by 2D TOCSY and 2D ^13^C–^1^H HSQC experiments. For sequence analysis, 2D NOESY experiments were carried out, which employed the anomeric signals of the residues A–D for compound **3** and A-E for compound **4** as starting points for the interpretations of the spectra. Taking literature data of ^1^H and ^13^C chemical shifts of *N*-glycans^7^ into account, the downfield chemical shifts of Man-B C-3 (δ 80.6) and C-6 (δ 65.9) confirmed the presence of 3,6-disubstituted Man-B residue of compound **3**. The anomeric regions of the 1D ^1^H NMR spectrum of the two compounds were similar except for an additional anomeric signal at δ_H_ 4.49 (residue E), which is attributed to a bisecting GlcNAc residue. The HSQC spectrum (Figures S3 and S5) showed downfield shifts for Man-B C-3 (δ 78.8), C-4 (δ 71.8), and C-6 (δ 65.3) typical for bisected *N*-glycan structures. Furthermore, the inter-residue NOESY cross-peak between GlcNAc-E H-1 (δ 4.49) and Man-B H-4 (δ 4.11) confirm the presence of additional E(1 → 4)B linkage in compound 4 (data not shown).

Next, the proteinaceous acceptors for transglycosylation were prepared (Figure 2B). Trastuzumab (**7**) was treated with wild-type Endo-S2 to yield **8**, which was incubated with BfFucH to remove the core-fucoside to afford the GlcNAc bearing mAb **9** (Figures 2B and S7–S9). The reactions were monitored by LC-MS, confirming product formation.

### Preparation of Glycoforms **10**, **11**, **12**, and **13**

**Figure 3:**
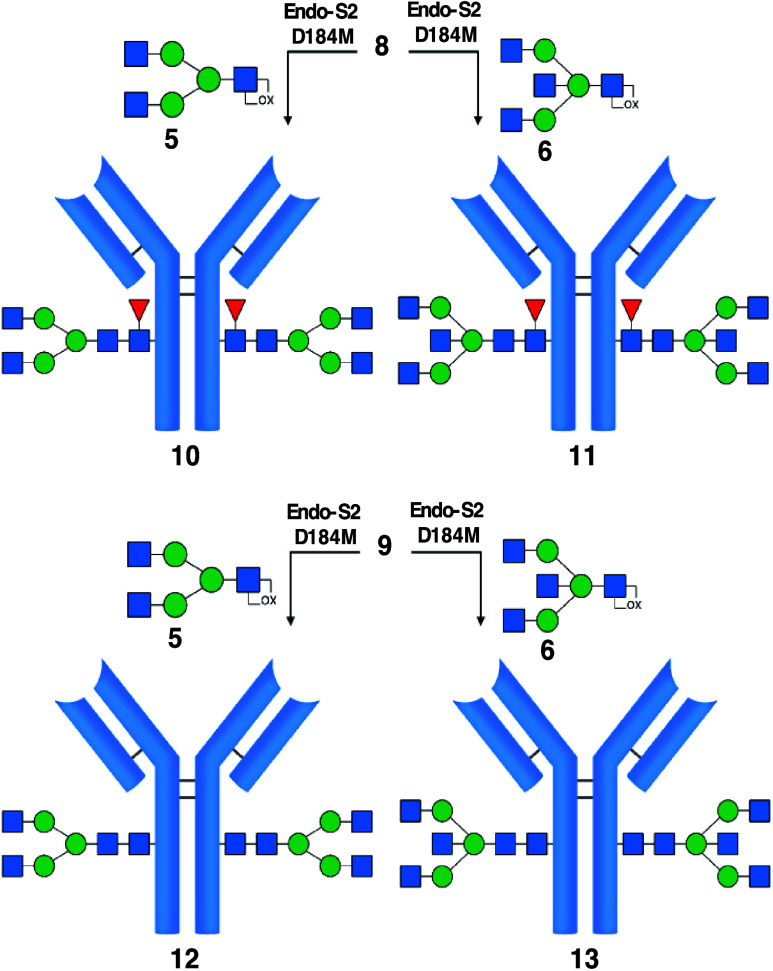
Glycosylation remodeling strategy to obtain glycoform **10**, **11**, **12**, and **13**. Endo-S2 D184M catalyzed the transglycosylation of A2-oxazoline **5** and A3B-oxazoline **6**. Reaction of **8** with **5** yielded A2-glycan bearing mAb **10**; reaction of **8** with **6** yielded A3B-glycan bearing mAb **11**; reaction of **9** with **5** yielded A2F-glycan bearing mAb **12**, and reaction of **9** with **6** yielded A3BF-glycan bearing mAb **13**. MS spectra confirming the structures after transglycosylation are found in Figures S10–S13.

Transglycosylation catalyzed by Endo-S2 D184M was carried out using either glycan-remodeled antibody **8** or **9** in combination with A2-oxazoline **5** and A3B-oxazoline **6** (Figure 3). Reactions of **8** and **5** yielded A2-glycan bearing mAb **10**; reaction of **8** and **6** yielded A3B-glycan bearing mAb **11**; reaction of **9** with **5** yielded A2F-glycan bearing mAb **12**, and, last, reaction of **9** with **6** yielded A3BF-glycan bearing mAb **13**. The progress of the reactions was monitored by LC-MS, and deconvolution of the spectra showed that despite the use of optimized reaction conditions for the transglycosylation, full conversion could not be reached in a single reaction (Figure S6). The transfer of the activated glycan was slightly better when the acceptor was the nonfucosylated mAb **9** compared to fucosylated mAb **8**, giving approximate conversions of 74 and 64%, respectively. The transglycosylation efficiency was independent of the type of glycan-oxazoline, as the use of A2- and A3B-oxazoline gave similar results. To improve the conversion rate, we applied other glycosynthases such as Endo-S D233Q^27^ and Endo-S D233Q/Q303L;^28^ however, the use of these enzymes did not offer better results, and the conversion rate was lower than for Endo-S D184M. To obtain homogeneous glycoforms, the incompletely modified trastuzumab variants were purified by protein A chromatography and resubmitted to transglycosylation. This approach yielded full conversions, resulting in the anticipated homogeneous trastuzumab glycoforms **10**, **11**, **12**, and **13**. The observed masses in the deconvoluted MS spectra of compounds **10**–**13** (Figures S10–S13) agreed with the theoretical masses as shown in Table S1. Immediately after reaching full conversion, the antibodies were purified by protein A chromatography, and the purity was confirmed by gel electrophoresis (Figure S14).

### mAbs Having a Bisecting GlcNAc Have Not Improved Affinity for Fcγ-Receptors

**Figure 4:**
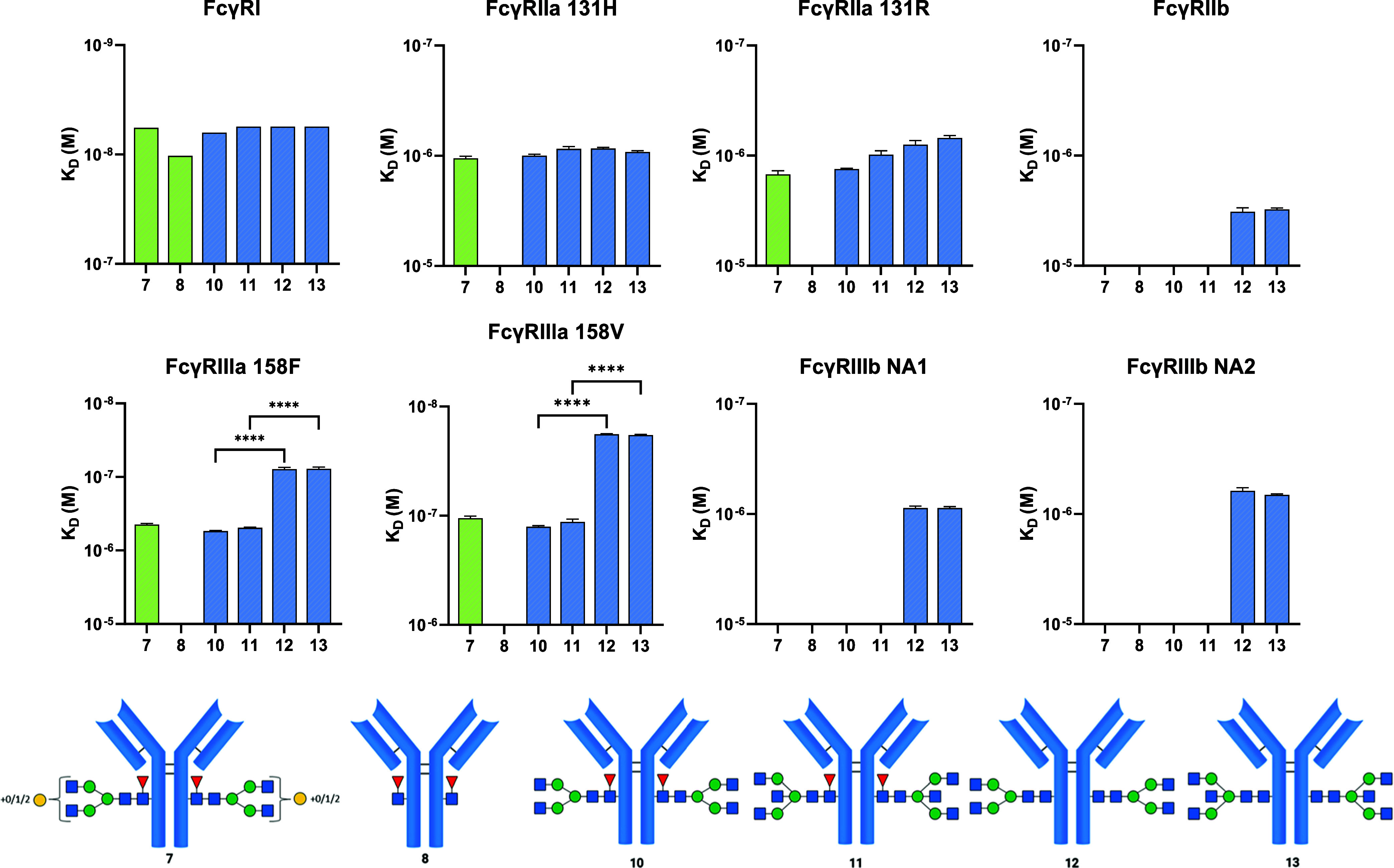
Characterization of the binding strength of variants to human FcγRs. *K*_d_ values for each variant are plotted as bar graphs, and error bars indicate scanning electron microscopy (SEM)-independent measurements. To determine significant differences between the affinity of the variants, we used a one-way ANOVA test, and significant differences are indicated with asterisks: ****p < 0.0001. Controls (green): mAbs **7** and **8**. Analytes (blue): No significant difference was observed between **10** and **11** and between **12** and **13**, meaning that there is no direct effect of the bisecting GlcNAc alone while core-fucosylation caused a 10-fold decrease in affinity for FcγRIIIa-158F and −158 V (comparing **10** and **12** or **11** and **13**). GlcNAc-Fuc-mAb **8** only engaged with FcγRI, indicating the necessity of a further expanded Fc-glycan for binding to the rest of the panel of FcγRs. Apparently, fucosylation obstructed binding to FcγRIIb and FcγRIIIb-NA1 and -NA2, as no binding was observed for **7**, **8**, **10**, and **11**. The control antibody **7** contains a small amount of an afucosylated glycan.

With the four trastuzumab variants **10**, **11**, **12**, and **13** in hand, we conducted SPR binding experiments to determine the affinity of the variants to a panel of human FcγRs.^29^ FcγRs, FcγRI, FcγRIIa-131H, FcγRIIa-131R, FcγRIIb, FcγRIIIa-158 V and -158F, and FcγRIIIb-NA1 and -NA2 were immobilized at 4 different concentrations on the sensor chip using either their His-tag or biotinylated Avitag. The FcγRIIa-variants are known to have different binding affinities for IgG2. FcγIIa-131H is the high-binding allele, and FcγIIa-131R is the low-binding variant. FcγRIIIa polymorphisms are known to result in a low-functioning receptor when phenylalanine is incorporated at residue 158 and a high-functioning receptor when valine is incorporated at residue 158. FcγRIIIb is expressed only by neutrophils, and FcγRIIIb-neutrophil antigen (NA)1/NA2 polymorphism influences phagocytosis after engaging with an IgG; hence, multiple polymorphisms are investigated.^30,31^ After immobilization of the FcγRs, the four mAbs were examined as analytes at concentrations ranging from 0.5 to 1000 nM. The outcome of the assessment of binding affinities, expressed as dissociation constants (*K*_d_), is shown in Figure 4. Unmodified mAb **7** and GlcNAc-Fuc bearing mAb **8** served as controls.

There was no significant difference between the trastuzumab variants in affinity for FcγRI, although the binding of mAb **8**, which has just a GlcNAc-Fuc core, was slightly weaker. Aglycosylated variant **8** did not bind to any other FcγR, clearly demonstrating the importance of an *N*-glycan for binding, which aligns well with previous reports.^32,33^ No differences in affinity of the variants for the receptor FcγRIIa-131H and FcγRIIa-131R were observed. Interestingly, variants **12** and **13** bound FcγRIIb, FcγRIIIb-NA1, and FcγRIIIb-NA2 with equal affinity, while the other fucosylated variants did not. Apparently, the mAbs’ core-fucosylation negates binding to FcγRIIb, FcγRIIIb-NA1, and FcγRIIIb-NA2. The affinity for FcγRIIIa-158F and FcγRIIIa-158V is greatly influenced by the absence of core-fucose, but bisecting GlcNAc does not appear to play a direct role as both **10** and **11** gave similar affinities. The same trend was observed for the binding of **10** and **11** for FcγRIIIa-158F and FcγRIIIa-158V, and in both cases, the binding is comparable to **7**, which is the wild-type variant consisting of a mixture of A2F, G1F, and G2F glycoforms (Table S1 and Figure S7). In conjunction with the core-fucosylated structures, in the mAbs without any core-fucose, in variants **12** and **13**, bisection did not improve nor attenuate binding affinity for FcγRIIIa-158F and FcγRIIIa-158V, which is in line with a previous report.^15^ Previous findings indicated that bisecting GlcNAc slightly increases FcγRIIIa binding; however, these studies were performed with IgG antibodies carrying unnatural bisected glycans lacking GlcNAc moieties at the α1,3- and α1,6-branching-mannosides.^16^ Antibodies having naturally occurring bisecting glycans, such as the A3B-glycan and A3BF-glycan presented in the current study, showed no improved binding to FcγRIIIa. Thus, the discrepancy with the previous study is likely due to differences in glycosylation at the branching mannosides.

Thus, bisecting GlcNAc alone does not cause an improvement of binding of the tested human FcγRs, while only core-fucosylation had a major influence on FcγR binding, especially for FcγRIIIa.

### FUT8 Cannot Modify A3B-Glycopeptide 15

Although bisecting GlcNAc does not directly affect the binding to FcγRs, several reports^10,13,14^ indicate that glycoengineered products from MGAT3 overexpressing cell lines influence ADCC activity. As described above, glycan analyses have shown that core-fucosylation of mAbs in MGAT3 overexpressing cells is reduced.^10,13^ To confirm that FUT8 is unable to fucosylate a bisected *N*-glycan, we created two acceptors, A2-glycopeptide **14** and A3B-glycopeptide **15**, to determine the kinetic parameters of fucose transfer using a GDP-Glo assay. It uncovered that FUT8 is highly active and can install a core-fucose on A2-glycopeptide **14** (Table 1), whereas no detectable activity for A3B-glycopeptide **15** was observed. The determined kinetic parameters of A2-glycopeptide **14** are in accordance with other reports^34−36^ and structural data on the FUT8/A2-glycopeptide complex.^34^ Thus, in an *in vitro* setting, FUT8 is unable to install a core-fucose when a bisecting GlcNAc moiety is present, and most likely, similar selectivities occur in the *trans* Golgi when glycans of glycoprotein are elaborated. Since overexpression of MGAT3 still results in some glycoforms with both bisecting GlcNAc and core-fucose, as was shown in glycomics studies,^11,13,37^ such structures must be produced by sequential enzymatic processing, first by core-fucosylation by FUT8, followed by modification by MGAT3.

**Table 1:**
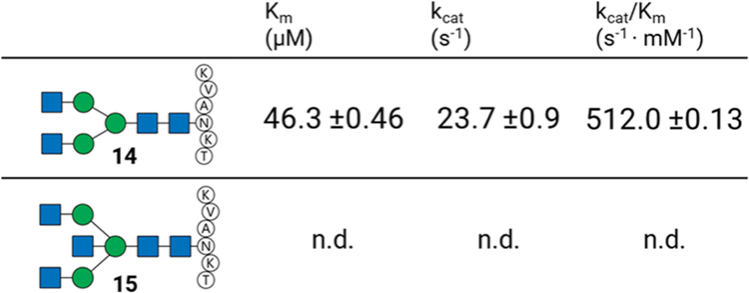
Kinetic Parameters of FUT8 for A2-glycopeptide **14** and A3B-glycopeptide **15**^a^

a GDP-Glo assay results of FUT8. Acceptors: A2-glycopeptide **14** and A3B-glycopeptide **15**. All reactions were conducted in triplicate, and values for *V*_max_ and *K*_m_ were determined using GraphPad Prism 9. Data are the mean of three replicates ± SEM n.d. = no fucosyl transfer by FUT8 detected.

## Conclusions

The preparation of well-defined glycoforms of antibodies has uncovered an interplay between core-fucosylation, galactosylation, and 2,6-sialylation of the *N*-glycan of the Fc-region of IgG antibodies.^12,38^ The preparation of well-defined glycoforms of IgG has also led to the identification of an optimal structure that is able to enhance the activities of antibodies for several diseases.^15^

The interplay between core-fucose and bisecting GlcNAc has not been studied in a systematic manner. Cell lines that coexpress MGAT3 have been used to develop mAbs with superior effector functions, and for example, an antineuroblastoma and an anti-CD20 mAb exhibit higher ADCC activities.^10,13,14^ In preclinical *in vivo* lymphoma models, the anti-CD20 mAb modified by bisecting GlcNAc outperformed the conventional anti-CD20 mAb (rituximab). In a phase I/II study, a group of 24 patients who had already received rituximab treatment were exposed to the anti-CD20 mAb with bisecting GlcNAc. It had a similar safety profile to rituximab, and promising efficacy in this difficult-to-treat patient population was observed.^39^ These observations indicate that a mAb produced in an MGAT3 overexpressing cell line has superior properties both *in vitro* and *in vivo*.

In this study, we prepared for the first time homogeneous glycoforms with variations in both core-fucose and bisecting GlcNAc. Structures with the A3BF-glycan have been identified in glycomics studies and, thus, are biologically relevant.^13,37^ It made it possible to investigate in a systematic manner whether bisecting GlcNAc can, through its back-fold conformation, modulate FcγR binding. It was found that bisecting GlcNAc does not affect the binding of both core-fucosylated mAbs with and without bisecting GlcNAc and that both variants performed equally well in FcγR binding assessments. Whether it is through cell engineering by means of knockout of the FUT8 gene or by indirectly restricting FUT8’s activity by overexpressing MGAT3, the focus should be to reduce core-fucosylation to acquire optimal FcγR binding. Overexpression of MGAT3 in antibody-producing cell lines results, however, in antibodies that are only partially modified by bisecting GlcNAc (50–70%) having still substantial core-fucosylation^11,13^ and, thus, is a less efficient approach to modulate FcγR binding.

## Experimental Procedures

### Preparation of A2-Glycan 3

Sialylglycopeptide (SGP, **1**) was extracted as previously described.^25^ White fluffy SGP (155.0 mg) was dissolved in 50 mM MOPS (pH 6.5, 3 mL) to which neuraminidase (*C. perfringens*, 40 μL, 1000 U, P0720, New England Biolabs) and galactosidase BgaA (300 μL, 1 mg mL^–1^) was added, and the reaction mixture was incubated for 18 h at 37 °C with gentle shaking. Progress of the reaction was monitored by LC-MS and MS spectra of compounds **1** and **2** and is found in Figure S1. Next, aqueous NaOAc (50 μL, 1 M) was added, and the pH was adjusted to 4.5. Endo-S2 WT (100 μL, 1 mg mL^–1^) was added, and the reaction mixture was further incubated overnight at 37 °C with gentle shaking. Another portion of Endo-S2 was added, and incubation was continued overnight to fully convert the start material into **3**. The reaction mixture was centrifuged over a Vivaspin 10 kDa MWCO filter, and the filtrate was lyophilized following P2 size-exclusion and semipreparative HPLC purification using a HILIC column, yielding 20.5 mg (34%) of A2-glycan **3** (Table 2).

**Table 2:** ^1^H and ^13^C NMR Chemical Shifts of A2-Glycan **3**, Recorded in D_2_O at 298 K^a^

Residue	H-1	H-2	H-3	H-4	H-5	H-6a/H6b	
	C-1	C-2	C-3	C-4	C-5	C-6a	CH_3_
→4)-α-d-GlcNAc	5.23	3.91	3.93	3.74	3.93	3.92/3.80	2.01/21.2
**A**_**α**_	90.3	53.7	69.4	80.1	69.6	60.4	
→4)-β-d-GlcNAc	4.74	3.72	3.73	3.74	3.57	3.87/3.79	2.07/22.3
**A**_**β**_	94.7	55.3	73.0	80.1	73.4	60.2	
→3,6)-β-d-Man-(1→	4.79	4.27	3.80	3.88	3.78	3.98	
**B**	100.4	70.2	80.6	69.6	73.3	65.9	
→2)-α-d-Man-(1→	4.93	4.13	3.92	3.51	3.63	3.90/3.65	
**C’**	97.0	76.5	69.4	67.2	73.2	61.6	
→2)-α-d-Man-(1→	5.13	4.21	3.92	3.53	3.64	3.90/3.65	
**C**	99.5	76.5	69.4	67.2	73.2	61.6	
β-d-GlcNAc-(1→	4.57	3.72	3.57	3.47	3.46	3.93/3.78	2.07/22.3
**D’**	99.5	55.5	73.4	69.9	75.8	60.6	
β-d-GlcNAc-(1→	4.57	3.72	3.57	3.47	3.46	3.93/3.78	2.07/22.3
**D**	99.5	55.5	73.4	69.9	75.8	60.6	

a MS and NMR spectra are found in Figure S2.

### Preparation of A3B-Glycan 4

To install the bisecting GlcNAc, A2-glycan **3** (7.8 mg, 7.0 μmol) was dissolved in 50 mM HEPES (pH 7.0), with 10 mM MnCl_2_ (500 μL) and UDP-GlcNAc (14 mg, ∼ 3 molar equiv, 11787900103, Roche), MGAT3 (150 μg) and calf intestine alkaline phosphatase (10 μL, 10 U, M1821, Promega) were added, and the mixture was incubated overnight at 37 °C with gentle shaking. The progress of the reaction was monitored by MS. The reaction mixture was centrifuged over a Vivaspin 10 kDa MWCO filter, and the filtrate was lyophilized followed by purification over a P2 size-exclusion column followed by semipreparative HPLC using a HILIC column, yielding 4.6 mg (50%) of (Table 3).

**Table 3:** ^1^H and ^13^C NMR Chemical Shifts of A3B-Glycan **4**, Recorded in D_2_O at 298 K^a^

Residue	H-1	H-2	H-3	H-4	H-5	H-6a/H6b	
	C-1	C-2	C-3	C-4	C-5	C-6a	CH_3_
→4)-α-d-GlcNAc	5.23	3.94	3.94	3.75	3.95	3.92/3.80	2.01/21.3
**A**_**α**_	90.5	53.6	69.6	79.5	69.9	60.4	
→4)-β-d-GlcNAc	4.74	3.73	3.74	3.74	3.57	3.87/3.79	2.08/22.3
**A**_**β**_	95.0	55.3	73.2	79.5	74.8	60.2	
→3,4,6)-β-d-Man-(1→	4.73	4.21	3.89	4.11	3.75	3.92	
**B**	100.4	70.5	78.8	71.8	72.5	65.3	
→2)-α-d-Man-(1→	5.02	4.17	3.87	3.51	3.65	4.00/3.66	
**C’**	97.9	76.1	69.6	67.2	73.2	61.9	
→2)-α-d-Man-(1	5.08	4.27	3.92	3.51	3.73	3.90/3.62	
**C**	100.1	76.5	69.5	67.2	73.2	61.6	
β-d-GlcNAc-(1→	4.57	3.72	3.61	3.48	3.43	3.90/3.8	2.08/22.2
**D’**	99.7	55.5	73.2	69.7	73.1	60.4	
β-d-GlcNAc-(1→	4.57	3.72	3.63	3.50	3.45	3.90/3.8	2.08/22.2
**D**	99.7	55.5	73.2	69.7	73.1	60.4	
β-d-GlcNAc-(1→	4.49	3.72	3.57	3.27	3.43	3.98/3.69	2.08/22.2
**E**	100.7	55.5	74.2	70.9	76.8	61.7	

a MS and NMR spectra are found in Figure S3.

### Preparation of A2-Oxazoline 5

To A2-glycan **3** (12.7 mg, 11.4 μmol) in water (1.0 mL), 2-chloro-1,3-dimethylimidazolinium chloride (39 mg, 20 molar equiv, 529249, Sigma-Aldrich) and EtN_3_ (95 μL, 60 molar equiv) were added, and the resulting mixture was left at 4 °C for 2 h. The progress of the reaction was monitored by LC-MS. The reaction mixture was directly loaded onto a Sephadex G10 column (150 cm diameter, 1.5 cm) using 0.001% EtN_3_ (pH 8.5) in water as eluent, run under gravity. Pure fractions were combined and lyophilized to afford 10.1 mg (81%) of A2-oxazoline **5** (Table 4).

**Table 4:** ^1^H and ^13^C NMR Chemical Shifts of A2-Oxazoline **5**, Recorded in D_2_O at 298 K^a^

Residue	H-1	H-2	H-3	H-4	H-5	H-6a/H6b	
	C-1	C-2	C-3	C-4	C-5	C-6a	CH_3_
→4)-GN-oxa	6.11	4.21	4.40	3.77	3.44	3.93/3.66	2.08/22.1
**A**	99.8	64.9	69.0	77.7	70.7	61.5	
→3,6)-β-d-Man-(1→	4.76	4.16	3.74	3.83	3.61	4.00/3.79	
**B**	101.2	70.0	80.2	65.6	74.1	65.5	
→2)-α-d-Man-(1→	4.95	4.16	3.90	3.53	3.66	3.93/3.65	
**C’**	96.4	75.9	69.2	67.1	72.6	61.4	
→2)-α-d-Man-(1→	5.13	4.21	3.92	3.52	3.76	3.93/3.65	
**C**	99.5	76.5	69.2	67.1	73.3	61.4	
β-d-GlcNAc-(1→	4.60	3.73	3.58	3.47	3.46	3.93/3.79	2.08/22.1
**D’**	99.3	55.1	73.1	69.7	75.6	60.4	
β-d-GlcNAc-(1→	4.57	3.71	3.57	3.47	3.46	3.93/3.78	2.08/22.1
**D**	99.5	55.1	73.1	69.7	75.6	60.4	

a MS and NMR spectra are found in Figure S4.

### Preparation of A3B-Oxazoline 6

To A3B-glycan **4** (4.6 mg, 3.5 μmol) in water (320 μL), 2-chloro-1,3-dimethylimidazolinium chloride (12 mg, 20 molar equiv, 529249, Sigma-Aldrich) and Et_3_N (29 μL, 60 molar equiv) were added, and the resulting mixture was incubated at 4 °C for 2 h. The progress of the reaction was monitored by LC-MS. The reaction mixture was directly loaded onto a Sephadex G10 column (150 cm, diameter 1.5 cm) and eluted with water containing 0.001% EtN_3_ (pH 8.5) under gravity. Fractions containing pure product were combined and lyophilized to afford 3.8 mg (88%) of A3B-oxazoline **6** (Table 5).

**Table 5:** ^1^H and ^13^C NMR Chemical Shifts of A3B-Oxazoline **6**, Recorded in D_2_O at 298 K^a^

Residue	H-1	H-2	H-3	H-4	H-5	H-6a/H6b	
	C-1	C-2	C-3	C-4	C-5	C-6a	CH_3_
→4)-GN-oxa	6.11	4.21	4.35	3.76	3.46	3.94/3.67	2.09/22.1
**A**	99.8	65.0	69.5	77.6	70.8	61.6	
→3,4,6)-β-d-Man-(1→	4.72	4.13	3.87	4.05	3.58	3.92	
**B**	101.0	70.1	78.4	71.7	74.1	65.3	
→2)-α-d-Man-(1→	5.04	4.21	3.88	3.53	3.67	4.00/3.66	
**C’**	97.0	75.7	69.4	67.1	73.2	61.7	
→2)-α-d-Man-(1→	5.08	4.27	3.93	3.49	3.73	3.90/3.62	
**C**	99.7	76.2	69.1	67.3	73.2	61.6	
β-d-GlcNAc-(1→	4.60	3.73	3.59	3.48	3.48	3.93/3.81	2.09/22.1
**D’**	99.2	55.2	73.2	69.6	75.7	60.3	
β-d-GlcNAc-(1→	4.57	3.73	3.62	3.50	3.48	3.93/3.81	2.09/22.1
**D**	99.7	55.2	73.2	69.6	75.7	60.3	
β-d-GlcNAc-(1→	4.49	3.73	3.59	3.29	3.44	4.00/3.71	2.09/22.1
**E**	100.6	55.8	74.0	70.8	76.4	61.5	

a MS and NMR spectra are found in Figure S5.

### Procedure for Preparing GlcNAc-Fuc Bearing mAb 8

Trastuzumab (gifted from ADC Therapeutics) was diluted to a concentration of 2 mg mL^–1^ in 50 mM NaOAc (pH 4.5). Per 100 μg of mAb, 1 μg of Endo-S2 WT was added, and the reaction mixture incubated for 2 h at 37 °C with gentle shaking. A weight ratio mAb/Endo-S2 WT of 100:1 was sufficient to yield the mAb with a GlcNAc-Fuc glycan. The progress of the reaction was monitored by LC-ESI-MS. The mAb was subjected to protein A purification prior to transglycosylation. The starting material and product MS spectrum are found in Figures S7 and S8, respectively.

### Procedure for Preparing GlcNAc Bearing mAb 9

mAb **8** was concentrated to 2 mg mL^–1^ in 100 mM TRIS (pH 7.5). BfFucH was added at a mAb/BfFucH weight ratio of 50:1, and the reaction mixture was incubated overnight at 37 °C with gentle shaking. The progress of the reaction was monitored using LC-ESI-MS. If the start material remained, an extra portion of BfFucH was added, followed by further incubation. In general, 3 additions of BfFucH and incubation for 3 nights yielded the complete defucosylated mAb. The mAb was subjected to protein A purification prior to transglycosylation. The product MS spectrum is found in Figure S9.

### Procedure for Preparing mAbs 10–13

To mAb 7 and **8** (2 mg mL^–1^ in 50 mM TRIS pH 7.5) was added either A2-oxazoline **5** or A3B-oxazoline **6**, followed by Endo-S2 D184M to generate mAbs **10**, **11**, **12**, and **13**. The weight ratio of mAb/Endo-S2 D184M was 30:1, and in each case, 50 molar equiv (to mAb) of glycan-oxazoline **5** or **6** was added. To generate mAb **10**, mAb **8** (3 mg) in 50 mM TRIS (pH 7.5, 1.5 mL), A2-oxazoline **5** (1.1 mg), and Endo-S2 D184M (100 μg) were incubated at 25 °C for a maximum of 3 h. Progress of the reaction was monitored by LC-ESI-MS taking a sample every 30 min. The combination of mAb **8** and A2-oxazoline **5** generated mAb **10**, the combination of mAb **8** and A3B-oxazoline **6** generated mAb **11**, the combination of mAb **9** and A2-oxazoline **5** generated mAb **12**, and the combination of mAb **9** and A3B-oxazoline **6** was used for generating mAb **13**. If any starting material remained, the mAb was subjected to protein A purification, and the reaction was repeated. The product MS spectra are found in Figures S10–S13. A Coomassie-stained SDS-PAGE gel of mAb **7**, **8**, and **10**–**13** is found in Figure S14.

### GDP-Glo Assay

For the determination of the FUT8 activity for the acceptor substrates glycopeptide **14** and glycopeptide **15**, a GDP-Glo Glycosyltransferase Assay (VA1093, Promega) was used. Briefly, 57.2 mg of SGP (**1**) was dissolved in 50 mM MOPS (pH 6.5, 1 mL), and neuraminidase (*C. perfringens*, 15 μL, 1000 U, P0720, New England Biolabs) and galactosidase BgaA (100 μL, 1 mg mL^–1^) were added and incubated for 18 h at 37 °C yielding A2-glycopeptide **14**. To half of the reaction mixture (∼500 μL), 500 mM MnCl_2_ (10 μL), UDP-GlcNAc (20 mg, ∼3 molar equiv) (11787900103, Roche), MGAT3 (100 μL, 1 mg mL^–1^), and calf intestine alkaline phosphatase (CIAP) (20 μL, 20 U, M1821, Promega) was added and incubated for 18 h at 37 °C yielding A3B-glycopeptide **15**. P2 size-exclusion and semipreparative HPLC purification using HILIC afforded **14** and **15** for the GDP-Glo assay.

The optimal enzyme concentration was determined by varying the FUT8 concentration in a range of 0.01–100 ng/μL while the concentrations of **14** and **15** were kept constant at 100 μM. The total reaction volume was 10 μL and consisted of the buffer component 50 mM TRIS (pH 7.5), 10 mM MnCl2, and 0.2 mM ultrapure GDP-Fuc (VA1097, Promega). The reaction mixtures were incubated for 1 h at 37 °C. Meanwhile, the GDP-Glo enzyme was diluted in the kit’s enzyme buffer, and the nucleotide detection reagent was added. After 1 h, GDP-Glo mix (10 μL) was added to the reaction mixture and incubated for another hour at 25 °C. Samples were transferred to a white 384-well plate, and luminescence was recorded. Once the optimal enzyme concentration for each of the substrates was established, it was fixed, and the substrate concentrations were varied in the range of 0.01–5 mM. All reactions were conducted in triplicate and evaluated using GraphPad Prism 9 (Prism).

### SPR Binding Studies

Affinity measurements to human FcγRs were performed by using an IBIS MX96 (IBIS Technologies) device and a Continuous Flow Microspotter (Wasatch Microfluidics). An array of 4 concentrations of a 3-fold dilution series of C-terminally biotinylated human FcγRs was captured simultaneously on a SensEye G-streptavidin sensor in 1× PBS containing 0.075% (v/v) Tween-80. All FcγRs were purchased from Sino Biological unless stated otherwise. The highest concentrations differed depending on the receptor and were 100 nM for hFcγRIIIa-158 V and hFcγRIIIb-NA1 (in-house), 30 nM for hFcγRIIIa-158 and hFcγRIIIb-NA2 (in-house), and 10 nM for hFcγRIIa-131H, hFcγRIIa-131R, and hFcγRIIb. A 2-fold dilution series of IgG variants thereof was injected ranging from 0.49 to 1000 nM in 1× PBS at pH 7.4 supplemented with 0.075% (v/v) Tween-80.

To measure the C-terminally His-tagged human FcγRI, 4 concentrations of a 3-fold dilutions series of C-terminally biotinylated anti-His mIgG1 (GenScript) were captured on a SensEye G-streptavidin sensor in 1× PBS containing 0.075% (v/v) Tween-80. Then, 40 nM hFcγRI was subsequently captured, and mAb variants thereof were titrated from 0.41 to 100 nM in a 3-fold dilution series in 1× PBS containing 0.075% (v/v) Tween-80. Regeneration of the sensor surface between the cycles was performed by an injection of 10 mM Gly-HCl at pH 2.1. Affinity calculations were carried out by performing an equilibrium analysis interpolating to an Rmax of 500 RU and fitting a 1:1 Langmuir binding model, assuming equilibrium to be reached after 360 s of IgG-containing analyte injections, as described previously.^40^ Analysis and calculations were performed using Scrubber Software Version 2 (BioLogic Software), Excel (Microsoft), and GraphPad Prism 9 (Prism).

## Supporting Information Available

General experimental procedures; Figures S1–S14, and Table S1 (PDF)
