# Supplementary material for: Bisecting N-Acetylglucosamine of the N-Glycan of Immunoglobulin G Does Not Affect Binding to Fc Gamma Receptors
Source: ACS Chem Biol. 2025 Feb 19;20(3):680–9. doi: 10.1021/acschembio.4c00807 (PMC11934091; doi:10.1021/acschembio.4c00807)
Supplement: Supplementary file 1 — cb4c00807_si_001.pdf [file cb4c00807_si_001.pdf]

Supporting Information

Bisecting *N*-Acetylglucosamine of the *N*-Glycan of Immunoglobulin G does not Affect Binding to Fc Gamma Receptors

Gerlof P. Bosman,[a] Inèz D. Stoof,[a] Hans P. Bastiaansen,[a] Linda Quarles van Ufford,[a] Justyna M. Dobruchowska,[a] Jan-Willem H. Langenbach,[a] Bhargavi M. Boruah,[b] Kelley W. Moremen,[b,c] Arthur E. H. Bentlage,[d] Suzanne N. Lissenberg-Thunnissen,[d] Gestur Vidarsson,[d,e] and Geert-Jan Boons[a,b,f,g] *

[a] Chemical Biology and Drug Discovery, Utrecht Institute for Pharmaceutical Sciences, Utrecht University, 3584 CG Utrecht, The Netherlands

[b] Complex Carbohydrate Research Center, University of Georgia, 315 Riverbend Road, Athens, GA 30602, United States

[c] Department of Biochemistry and Molecular Biology, The University of Georgia, Athens, GA 30602, United States

[d] Department of Experimental Immunohematology, Sanquin Research, Amsterdam, The Netherlands

[e] Department of Biomolecular Mass Spectrometry and Proteomics, Utrecht University, Utrecht, The Netherlands

[f] Bijvoet Center for Biomolecular Research, Utrecht University, 3584 CG Utrecht, The Netherlands

[g] Department of Chemistry, The University of Georgia, Athens, GA 30602, United States

* Corresponding author. Email: g.j.p.h.boons@uu.nl, gjboons@ccrc.uga.edu

Table of contents

General Experimental Procedures S3

Referencess S6

Figure S1. LC-MS spectra SGP 1 and A2-glycopeptide **2** S7

Figure S2. LC-MS and ^{1}H NMR and 2D ^{13}C-^{1}H HSQC spectra A2-glycan **3** S8

Figure S3. LC-MS and ^{1}H NMR and 2D ^{13}C-^{1}H HSQC spectra of A3B-glycan **4** S9

Figure S4. LC-MS and ^{1}H NMR and 2D ^{13}C-^{1}H HSQC spectra A2-oxazoline **5** S10

Figure S5. LC-MS and ^{1}H NMR and 2D ^{13}C-^{1}H HSQC spectra of A3B-oxazoline **6** S11

Theoretical masses vs observed masses Figure S7-S13 .... S12

Table S1. Theoretical masses versus observed masses S13

Figure S6. Initial attempts transglycosylation S14

Figure S7. Deconvoluted MS spectrum mAb **7** S16

Figure S8. Deconvoluted MS spectrum mAb **8** S17

Figure S9. Deconvoluted MS spectrum mAb **9** S18

Figure S10. Deconvoluted MS spectrum mAb **10** S19

Figure S11. Deconvoluted MS spectrum mAb **11** S20

Figure S12. Deconvoluted MS spectrum mAb **12** S21

Figure S13. Deconvoluted MS spectrum mAb **13** S22

Figure S14. SDS-PAGE gel - Coomassie stain of mAb **7, 8, 10-13** S23

General Experimental Procedures

Expression of BgaA, BfFucH, Endo-S2 WT and Endo-S2 D184M in *E. coli.* Endoglycosidases Endo-S2 wildtype[1] (WT) and Endo-S2 D184M[2] from *S. pyogenes* (AA38-843) (GenBank: AGU16855.1) were cloned in a custom vector based on pET11a, with a N-terminal STREP-tag (WSHPQFEK), a superfolder GFP (GenBank: AAA27721.1 + S30R/Y39N/N105T/Y145F/I171V/A206V) and a spacer (GGGSGGGSGGS), and a C-terminal HIS-tag (HHHHHHHH). The fucosidase BfFucH[3] (AA21-434, GenBank: CAH08937.1) was subcloned in pET47b+ (Acc65I/XhoI). The galactosidase BgaA (AA2-2221) (GenBank: AAK99369.1) was cloned in a custom vector based on pET11a with a N-terminal STREP-tag (WSHPQFEK), a mCherry (Uniprot D1MPT3 + N8D/K199N/T200V/D201N) and a spacer (GGGSGGGSGGS), and a C-terminal HIS-tag (HHHHHHHH). BL21(DE3) (C2527H, New England Biolabs) cells were transformed with each of the individual vectors, and plated on a 2xYT agar (BP97432, Fisher Bioreagents) plate with ampicillin (100 µg/mL; 14417, Cayman Chemical) or kanamycin (50 µg/mL; 11815024, Gibco). On the next day, a colony was picked and expanded to a cell culture volume of 500 mL antibiotic containing 2xYT medium (X966, Carl Roth) at 37 °C. The cells were grown for 3-5 h, until the enzyme production was induced at OD600 = 0.6 with isopropyl β-D-1-thiogalactopyranoside (IPTG; R0393, Thermo Scientific) at a final concentration of 1 mM. After induction, the cells were cultured overnight at 20 °C. Then, the cells were pelleted at 3000 x *g*, resuspended in lysis buffer (TBS; Tris 25 mM, NaCl 150 mM, pH 7.5) with 1 mg/mL lysozyme (62971, Sigma-Aldrich) and 0.1% triton X-100 (T8787, Sigma-Aldrich)) at 5% of the original culture volume. The resuspended cells were incubated at room temperature for 1 h and then sonicated. Sonication was performed using a Bandelin Sonopuls HD2200, with a MS73 probe, at an amplitude of 50%, 3 times 10 s on, 10 s off. supernatant was obtained by removal of cell debris by centrifugation at 10000 x *g*. The supernatant with the protein of interest was further purified by affinity chromatography and size-exclusion chromatography.

Expresssion of hGnT-III and hFUT8 in HEK293F Suspension Cells. The luminal portion of the enzyme hGnT-III (AA24-533) (GenBank CAG30405.1) and hFUT8 (AA31-575) (GenBank: AAB92372.2) were cloned into a so-called pGen2-vector resulting in a fusion protein of a N-terminal HIS-tag (HHHHHHHH), a superfolder GFP, a TEV protease site (ENLYFQG) and the enzyme of interest.[4] Both hGnT-III and hFUT-8 were expressed through transient transfection in wild type HEK293F cells (Freestyle 293F). Briefly, cells were maintained in suspension culture at 1-3 x 10^6 cells/mL in a humidified CO_2 shaker incubator (37 °C, 150 rpm). Transient transfection was performed at a cell density of 3-3.5 x 10^6 cells/mL in HEK-TF medium (861-0001, Xell) supplemented with GlutaMAX (35050061, Gibco) and Pen-Strep (15070063, Gibco) using predissolved, sterile, transfection grade, linear polyethylenimine (PEI) 25000 g/mol (23966, Polysciences), at a concentration of 9 µg/mL and a vector concentration of 4 µg/mL by directly, dropwise addition of the solutions to the culture medium. The day after the transfection the suspension culture was diluted 1:1 with HEK-TF

medium. Right after that, predissolved, sterile valproic acid (VPA) (P4542, Sigma-Aldrich) to a final concentration of 2.2 mM was added. Feed HEK-FS (871-0001, Xell) supplemented with GlutaMAX and Pen-Strep was added at day 1, 3, and 4 post-transfection at 5%, 7.5%, and 10% of the total culture volume respectively. On day 6 the supernatant was collected by centrifugation at 3000 *xg* at 4 °C for 20 min. After centrifugation, the supernatant was filtered over a 3 µm acetate filter to remove any residual cells and debris. The HEK293F cell-free supernatant containing the enzyme of interest was purified using a two-step purification procedure, first by Ni-NTA purification, followed by size-exclusion chromatography.

General Procedure for Ni-NTA Purification of *E. coli* and HEK Produced Enzymes. The enzymes BgaA, BfFucH, Endo-S2 WT, Endo-S2 D184M, hGnT-III, and hFUT8 were purified using Ni-NTA affinity chromatography. The clarified supernatant was loaded onto a 4 mL gravity flow Ni-NTA column (17-5318-01, GE healthcare), capable of binding the HIS-tagged protein. A standard buffer consisting of 50 mM TRIS-HCl and 250 mM NaCl pH 8 was made. Imidazole was added to the standard buffer at concentrations of 20 mM, 50 mM or 250 mM to make wash 1, wash 2, and elution buffer (re-adjusted to pH 8 if needed) respectively. Once washed with 10 column volumes (CV) of wash 1, 10 CV of wash 2, the enzyme of interest was eluted in 3 CV of elution buffer.

General Procedure for STREP-tag Purification of *E. coli* Expressed Enzymes. Through the STREP-tag the enzymes BgaA, BfFucH and Endo-S2 WT and Endo-S2 D184M were purified using Strep-Tactin Superflow resin (2-1206-025, IBA Lifesciences). The eluate from the Ni-NTA column was directly loaded onto a 4 mL gravity flow Strep-Tactin column. Elution buffer was prepared by addition of 2.5 mM desthiobiotin (D1411, Sigma-Aldrich) to wash buffer (100 mM TRIS, 150 mM NaCl, 1 mM EDTA, pH 8). Once the sample was loaded, unbound material was removed from the resin by washing with 10 CV of wash buffer, followed by elution of the protein of interest with 3-5 CV of elution buffer. Fractions of 2 mL were collected, pure fractions were pooled, concentrated, and stored.

General Procedure for SEC Purification of *E. coli* and HEK Produced Enzymes. Size exclusion chromatography was used to further purify the enzymes BgaA, BfFucH, Endo-S2 WT, Endo-S2 D184M, hGnT-III and hFUT8 after Ni-NTA or the STREP-tag purification. The eluate from either the Ni-NTA or the STREP-tag purification was concentrated using a Vivaspin 6 filter 10 kDa MWCO (VS0602, Sartorius) and further purified on a TBS (25 mM TRIS, 150 mM NaCl, pH 7.5) equilibrated Superdex 200 Increase 10/300 GL column (28990944, GE healthcare) attached to a Shimadzu Nexera system using a flow of 0.45 mL/min collecting one fraction each minute. Detection of protein be done at UV absorbance of 280 nm. Fractions that contained pure enzyme were pooled, concentrated, aliquoted and stored at -20 °C in TBS containing 10% glycerol.

Protein A Purification of mAbs. For purification of the glyco-remodelled mAbs, a gravity flow Protein A resin column was used. The Protein A Sepharose CL-4B resin powder (GE17-0780-01, Cytiva) was left to swell for 1 h in TBS before pouring into the empty gravity flow column. In general, 2 mL of swollen resin was used to purify 2-10 mg mAb. Once the column was poured, the resin bed was equilibrated by a wash of 10 CV of TBS (25 mM TRIS, 150 mM NaCl, pH 7.5). Then, the protein mixture consisting of the mAb, glycosyl hydrolases and transferases, and other small molecules was loaded after adjusting the buffers pH to 7-8. Subsequently, the resin bed was washed with 10 CV of TBS, followed by elution with 10 CV of 100 mM glycine buffer pH 3 in fractions of 2 mL. Usually, the mAb was eluted in fraction 2-5 (after 2-5 CV). The pH of the eluate (10 mL) was directly neutralized by adding 200 µL TRIS 1 M pH 8.5 and was then concentrated and buffer exchanged to ultrapure water using a 30 kDa MWCO Amicon Ultra-4 Centrifugal Filter (UFC8030, Millipore).

General Procedure for HILIC-MS Purification of Compounds 3, 4, 14, and 15. Prior HILIC purification of compound 3, 4 and 14 and 15, the glycans were purified using Biogel P-2 (1504118, Bio-Rad) size-exclusion chromatography. The lyophilized glycans **3** and **4** and glycopeptides **14** and **15**, free of proteinaceous content, were dissolved in 1 mL ultrapure water. The column dimensions were 150 cm with a diameter of 3 cm and was run with 50 mM NH_4HCO_3 buffer (pH ~8) under gravity flow. Fractions of 2 mL were collected, pure fractions were combined, lyophilized and further processed. For semipreparative purification of the P2 purified glycans/glycopeptides, a Shimadzu Nexera system with a Xbridge BEH Amide OBD column, 130Å, 5 µm, 10x250 mm (186006602, Waters) that was run with a linear gradient of 90% to 50% B vs A in 90 min at room temperature (A: 90% ultrapure water, 10% acetonitrile, 10 mM NH_4HCOO pH 4.5, B: 90% acetonitrile, 10% ultrapure water, 100 mM NH_4HCOO pH 4.5, at a flow rate of 3 mL/min was used. Fractions of 3 mL were collected, fractions that contained the pure product were combined and lyophilized. Glycans were detected by MS, to do so, the Shimadzu Nexera system was coupled to a Bruker MicroTOF via a splitter (500:1 – fraction collector:MS), with source settings; end plate offset -500 V, capillary voltage of -3500 V, nebulizer gas pressure was 25 psi and dry gas of 200 °C with a flow 6 L/min was pumped into the chamber.

NMR Analysis. Resolution-enhanced 1D/2D 600-MHz ^{1}H NMR spectra and 150-MHz ^{13}C NMR spectra were recorded on a Bruker Avance Neo spectrometer equipped with a TCI Prodigy CryoProbe™ at a probe temperature of 298 K. Prior to analysis, samples were exchanged twice in D_2O (99.9 atom % D, Cambridge Isotope Laboratories, Inc., Andover, MA) with intermediate lyophilization, and then dissolved in 0.5 mL D_2O. Chemical shifts (δ) are expressed in ppm by reference to internal acetone (δ 2.225 for ^{1}H and 31.07 for ^{13}C). Suppression of the HOD signal was achieved by applying a WEFT pulse sequence for 1D experiments and by a pre-saturation of 1 sec during the relaxation delay in 2D experiments. 2D TOCSY spectra were recorded using an MLEV-17 mixing sequence with spin-lock times of 40–150 ms. 2D NOESY experiments were performed with a mixing time of 300 ms. Natural

abundance 2D ^{1}H-^{13}C HSQC experiments were recorded without decoupling during acquisition of the ^{1}H FID.

LC-ESI-MS Analysis of mAbs 7-13. For analysis of the mAb Fc-glycan, samples were pretreated with the enzyme IdeS. This enzyme cleaves below the hinge region (PAPELLG|GPSV), resulting in a single Fc fragment with the glycan at N297 of ~25 kDa. Exact theoretical masses are listed in Table S1. To monitor the reaction progress, 1 µL TRIS 1 M pH 8.5 was added to a sample of 10 µg of mAb to adjust the pH ~7.5. Next, 10 units IdeS (A0-FR1, Genovis) were added and incubated at 37 °C for 30 min. A Vivaspin 500 10 kDa MWCO spin filter (VS0102, Sartorius) was used to exchange the reaction mixture to ultrapure water, prior MS analysis. Samples (1 µL) were injected with an Agilent 1290 Infinity LC equipped with an Acquity 300Å 1.7 µm, C18 2.1 x 50 mm column (186003685, Waters) over a linear gradient of 20-40% B *vs.* A in 7 min. at 70 °C (A: 99.9% ultrapure water, 0.1% formic acid, B: 70% isopropanol, 20% acetonitrile, 9.9% ultrapure water, 0.1% formic acid) with a flow rate of 0.3 mL/min. The detector was an Agilent 6560 Ion Mobility LC/Q-TOF only using the Q-TOF functionality. The Q-TOF source settings were optimized for glycoprotein detection; the gas temperature was 350 °C and the drying gas flow was set at 8 L/min, the pressure of the nebulizer gas was 45 psig, sheath gas of 400 °C with a flow of 11 L/min was pumped into the chamber. The fragmentor was set at 380 V, the capillary voltage was 5500 V and the nozzle voltage was 2000 V. Post-run analysis and deconvolution was performed using Agilent's Bioconfirm software.

References

(1) Sjögren, J.; Struwe, W. B.; Cosgrave, E. F. J.; Rudd, P. M.; Stervander, M.; Allhorn, M.; Hollands, A.; Nizet, V.; Collin, M. EndoS2 Is a Unique and Conserved Enzyme of Serotype M49 Group A Streptococcus That Hydrolyses N-Linked Glycans on IgG and α1-Acid Glycoprotein. *Biochem J* 2013, *455* (1), 107–118. DOI:10.1042/BJ20130126

(2) Li, T.; Tong, X.; Yang, Q.; Giddens, J. P.; Wang, L. X. Glycosynthase Mutants of Endoglycosidase S2 Show Potent Transglycosylation Activity and Remarkably Relaxed Substrate Specificity for Antibody Glycosylation Remodeling. *J Biol Chem* 2016, *291* (32), 16508–16518. DOI:10.1074/jbc.M116.738765

(3) Tsai, T. I.; Li, S. T.; Liu, C. P.; Chen, K. Y.; Shivatare, S. S.; Lin, C. W.; Liao, S. F.; Lin, C. W.; Hsu, T. L.; Wu, Y. T.; Tsai, M. H.; Lai, M. Y.; Lin, N. H.; Wu, C. Y.; Wong, C. H. An Effective Bacterial Fucosidase for Glycoprotein Remodeling. *ACS Chem Biol* 2017, *12* (1), 63–72. DOI:10.1021/acschembio.6b00821

(4) Moremen, K. W.; Ramiah, A.; Stuart, M.; Steel, J.; Meng, L.; Forouhar, F.; Moniz, H. A.; Gahlay, G.; Gao, Z.; Chapla, D.; Wang, S.; Yang, J. Y.; Prabhakar, P. K.; Johnson, R.; Rosa, M. Dela; Geisler, C.; Nairn, A. V.; Seetharaman, J.; Wu, S. C.; Tong, L.; Gilbert, H. J.; Labaer, J.; Jarvis, D. L. Expression System for Structural and Functional Studies of Human Glycosylation Enzymes. *Nat Chem Biol* 2018, *14* (2), 156–162. DOI:10.1038/nchembio.2539

Figure S1. LC-MS spectra SGP 1 and A2-glycopeptide 2

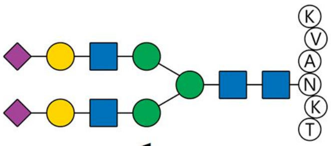

1

2865.8 g/mol

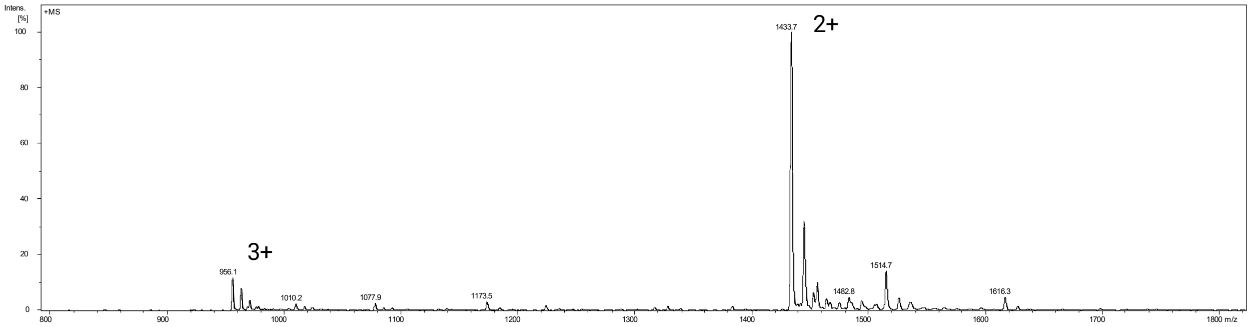

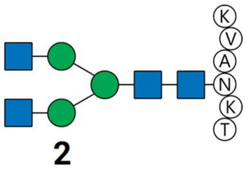

2

1959.0 g/mol

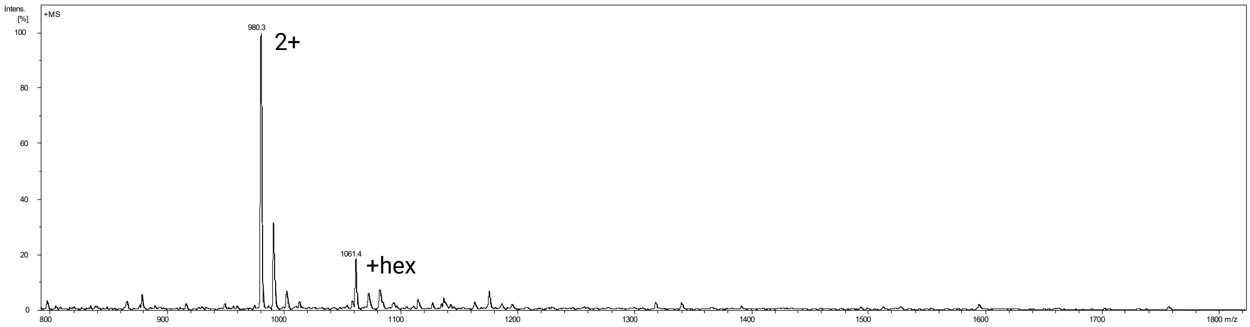

Figure S2. LC-MS and ^{1}H NMR and 2D ^{13}C-^{1}H HSQC spectra A2-glycan 3

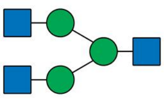

1114.0 g/mol

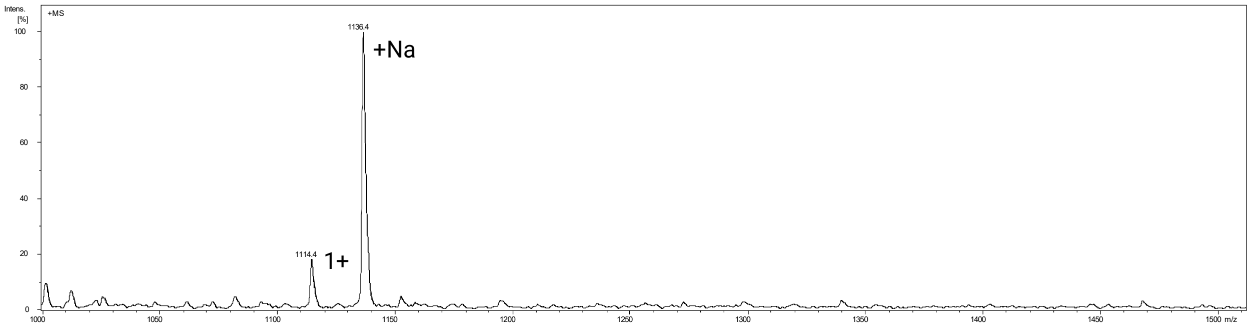

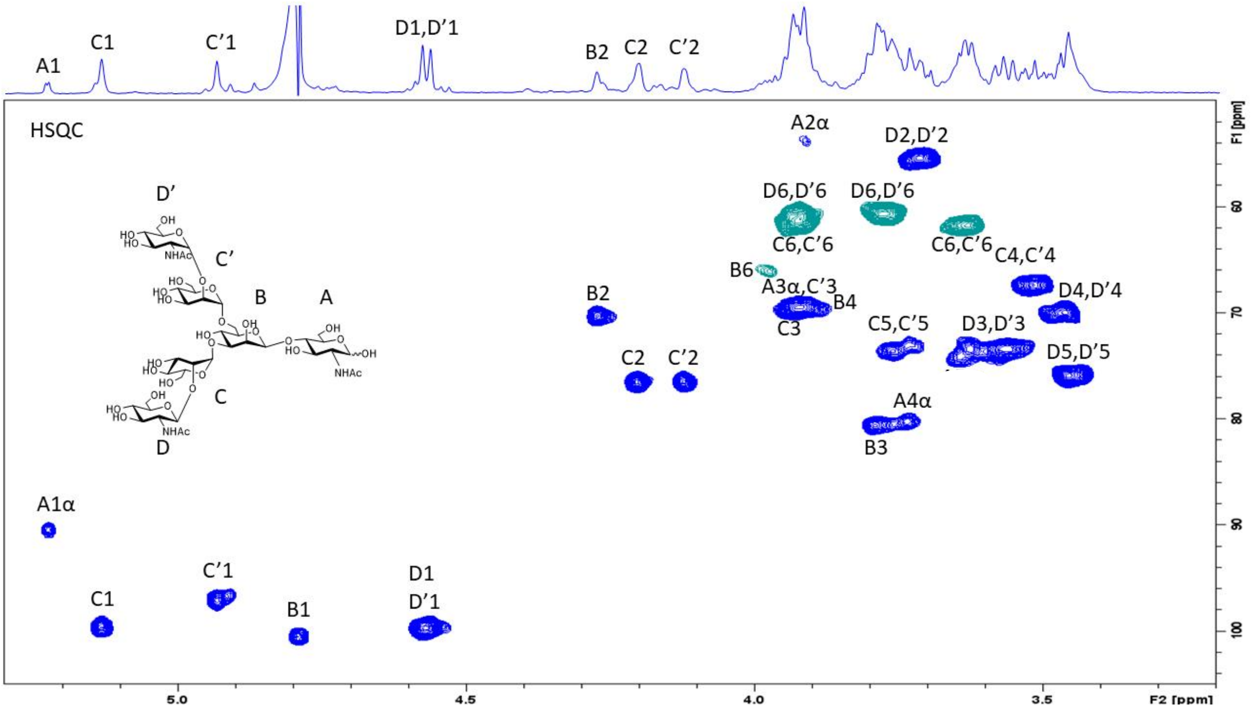

600 MHz 1D ^{1}H NMR and 2D ^{13}C-^{1}H HSQC spectra of A2-glycan **3**, recorded at 298K in D_2O

Figure S3. LC-MS and ^{1}H NMR and 2D ^{13}C-^{1}H HSQC spectra of A3B-glycan 4

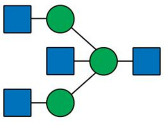

4

1317.2 g/mol

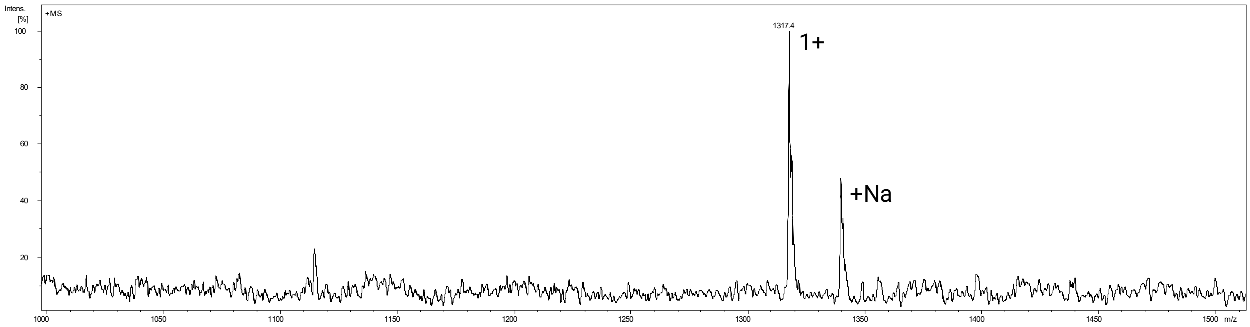

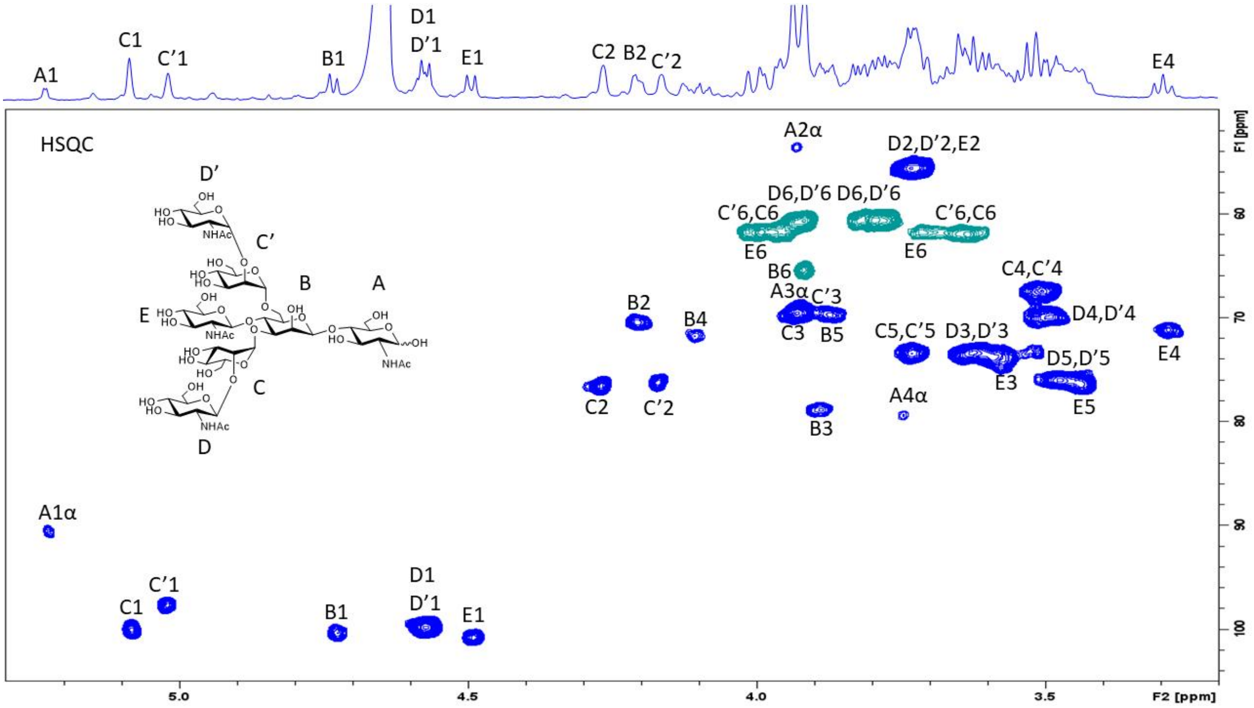

600 MHz 1D 1H NMR and 2D 13C-1H HSQC spectra of A3B-glycan **4**, recorded at 298K in D_2O

Figure S4. LC-MS and ^{1}H NMR and 2D ^{13}C-^{1}H HSQC spectra A2-oxazoline 5

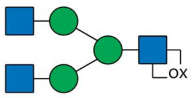

5

1096.0 g/mol

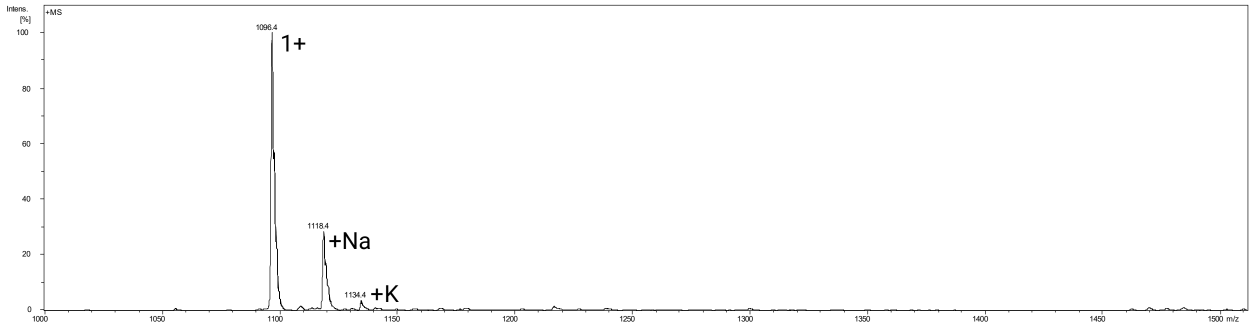

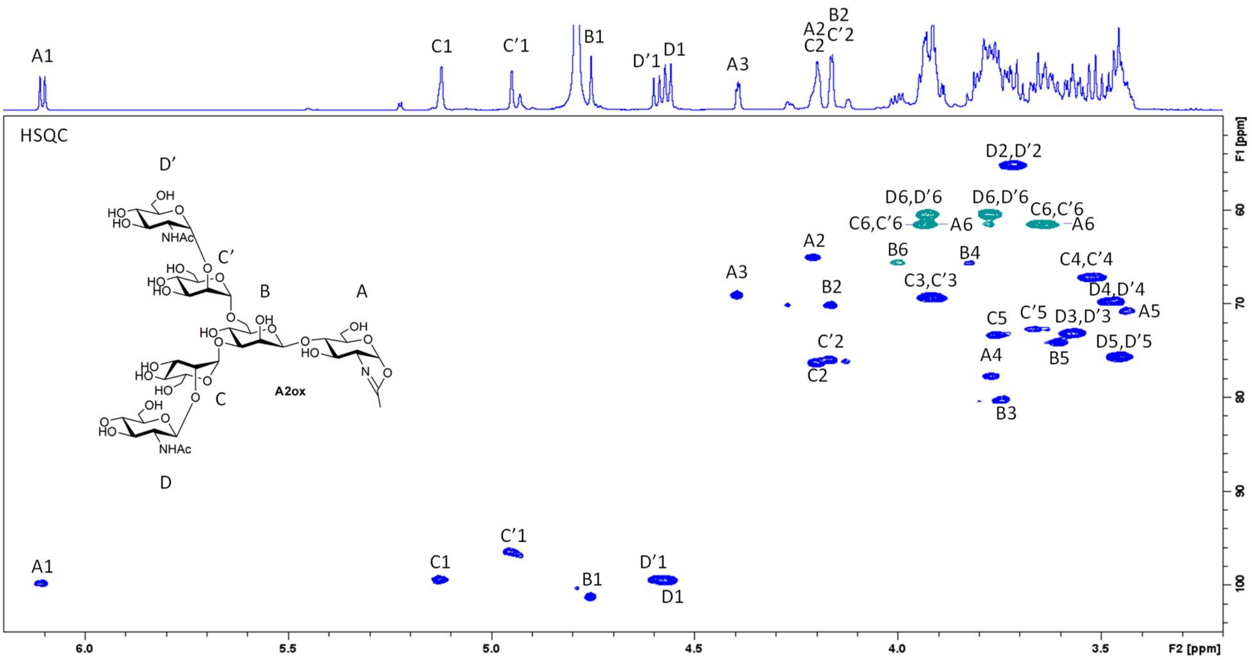

600 MHz 1D ^{1}H NMR and 2D ^{13}C-^{1}H HSQC spectra of A2-oxazoline **5**, recorded at 298K in D_2O

Figure S5. LC-MS and ^{1}H NMR and 2D ^{13}C-^{1}H HSQC spectra of A3B-oxazoline 6

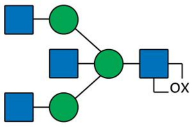

6

1299.2 g/mol

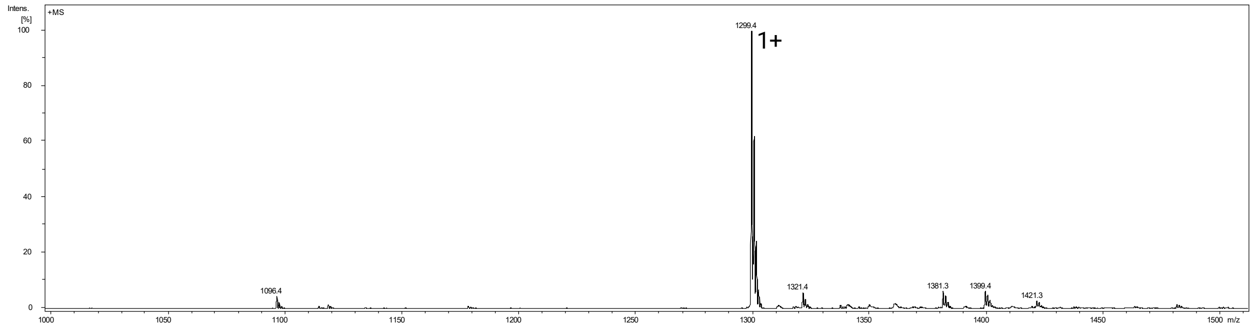

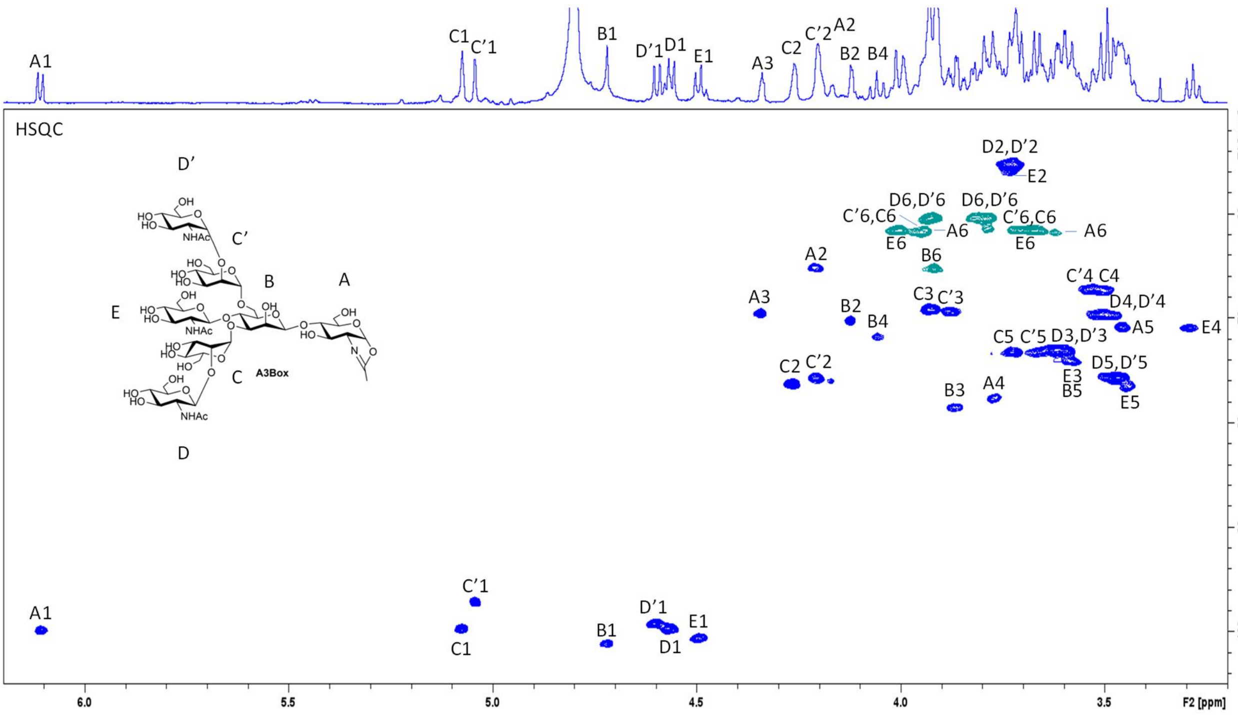

600 MHz 1D ^{1}H NMR and 2D ^{13}C-^{1}H HSQC spectra of A3B-oxazoline **6**, recorded at 298K in D_2O

Theoretical masses vs observed masses Figure S7-S13

For analysis of the mAb Fc-glycan, samples were pretreated with the enzyme IdeS. This enzyme cleaves below the hinge region (PAPELLG|GPSV), resulting in a single Fc fragment with the glycan at Asn-297 of about ~25 kDa, as shown in the example below.

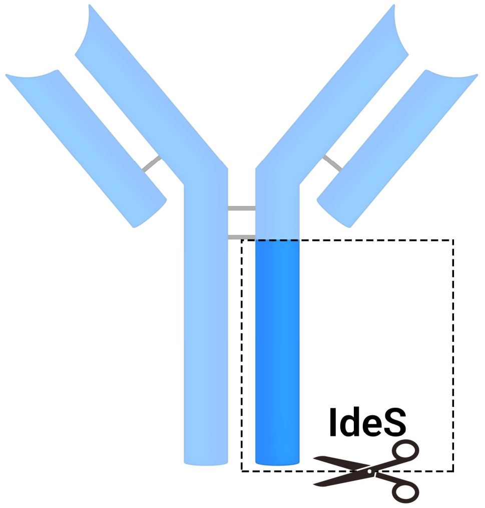

Trastuzumab Fc sequence after IdeS cleavage. Molecular weight; 23790.93 g/mol

```
                                                    230        240
                                                              GPSV
       250        260        270        280        290        300
FLFPPKPKDT LMISRTPEVT CVVVDVSHED PEVKFNWYVD GVEVHNAKTK PREEQYNSTY
       310        320        330        340        350        360
RVVSVLTVLH QDWLNGKEYK CKVSNKALPA PIEKTISKAK GQPREPQVYT LPPSREEMTK
       370        380        390        400        410        420
NQVSLTCLVK GFYPSDIAVE WESNGQPENN YKTTPPVLDS DGSFFLYSKL TVDKSRWQQG
       430        440
NVFSCSVMHE ALHNHYTQKS LSLSPG
```

Table S1. Theoretical masses versus observed masses

Trastuzumab (IdeS treated)				
#	glycan	theoretical	observed	comments
	no glycan	23790.9		
7	unmodified	25234.9	25234.5	A2F
			25397.6	G1F
			25559.1	G2F
8	GlcNAc-Fuc	24138.9	24138.7	5-10% GlcNAc
9	GlcNAc	23992.9	23993.1	
10	A2	25088.9	25088.7	
11	A3B	25290.9	25291.9	
12	A2F	25234.9	25234.3	
13	A3BF	25436.9	25438.3	

Figure S6. Initial attempts transglycosylation

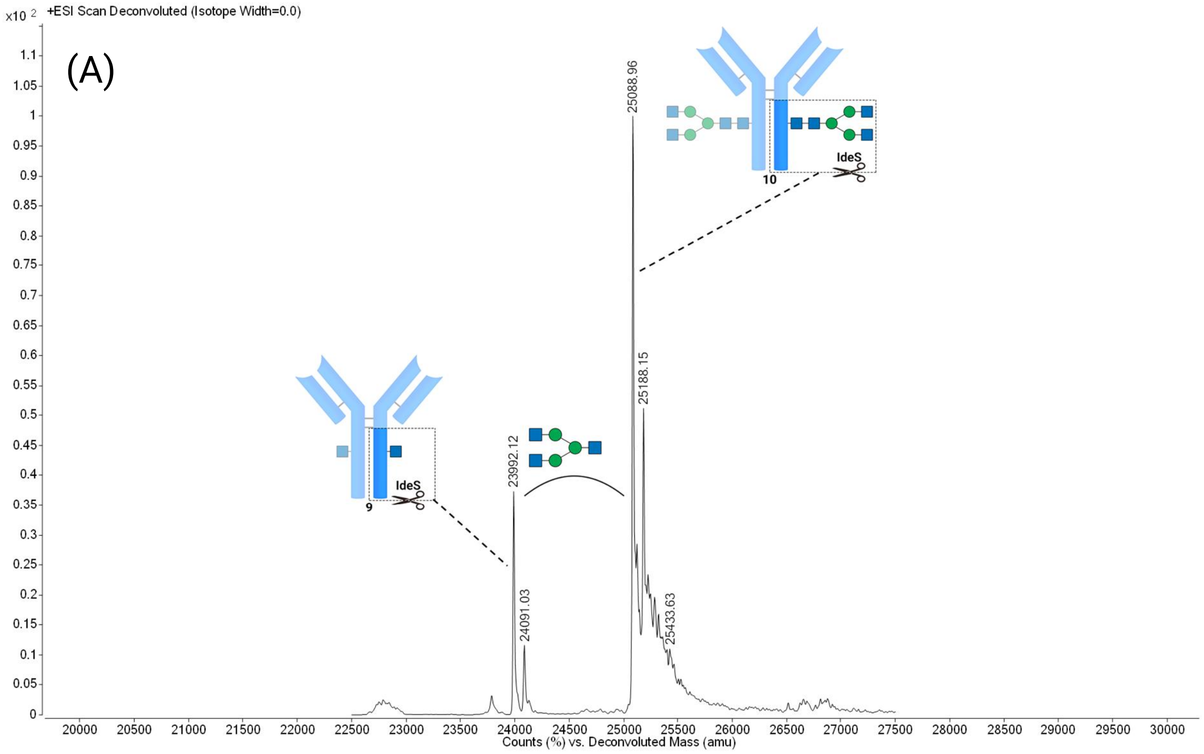

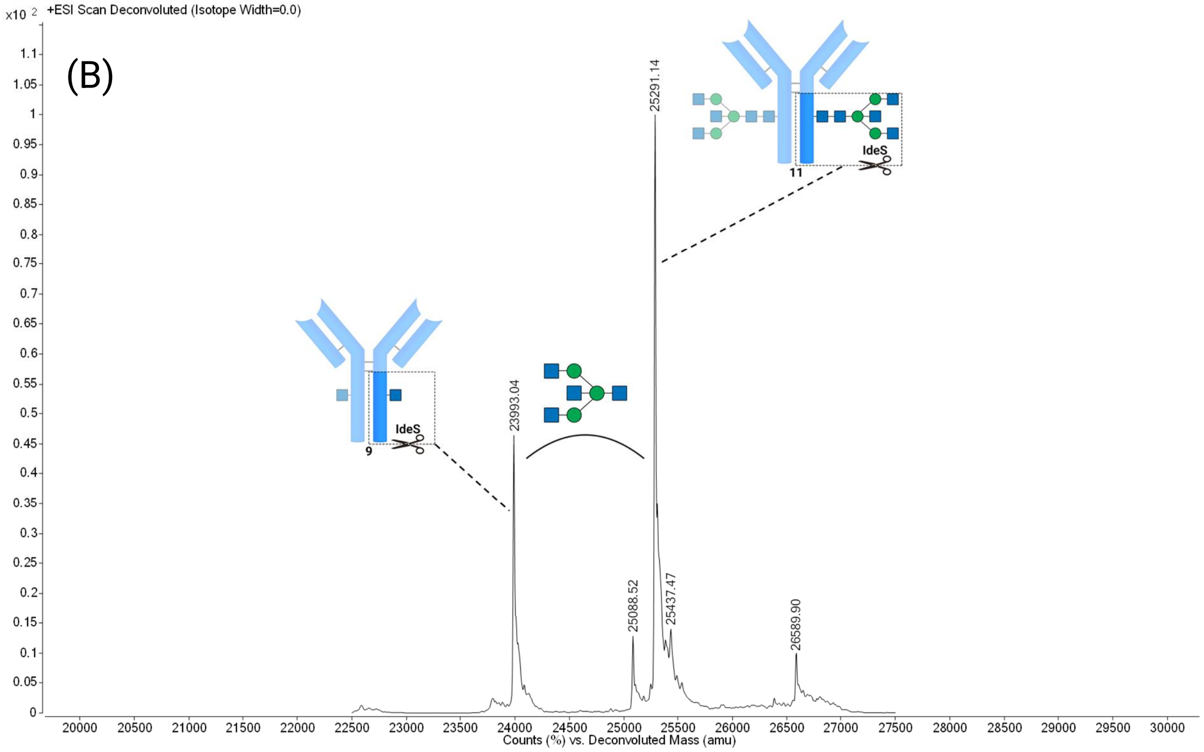

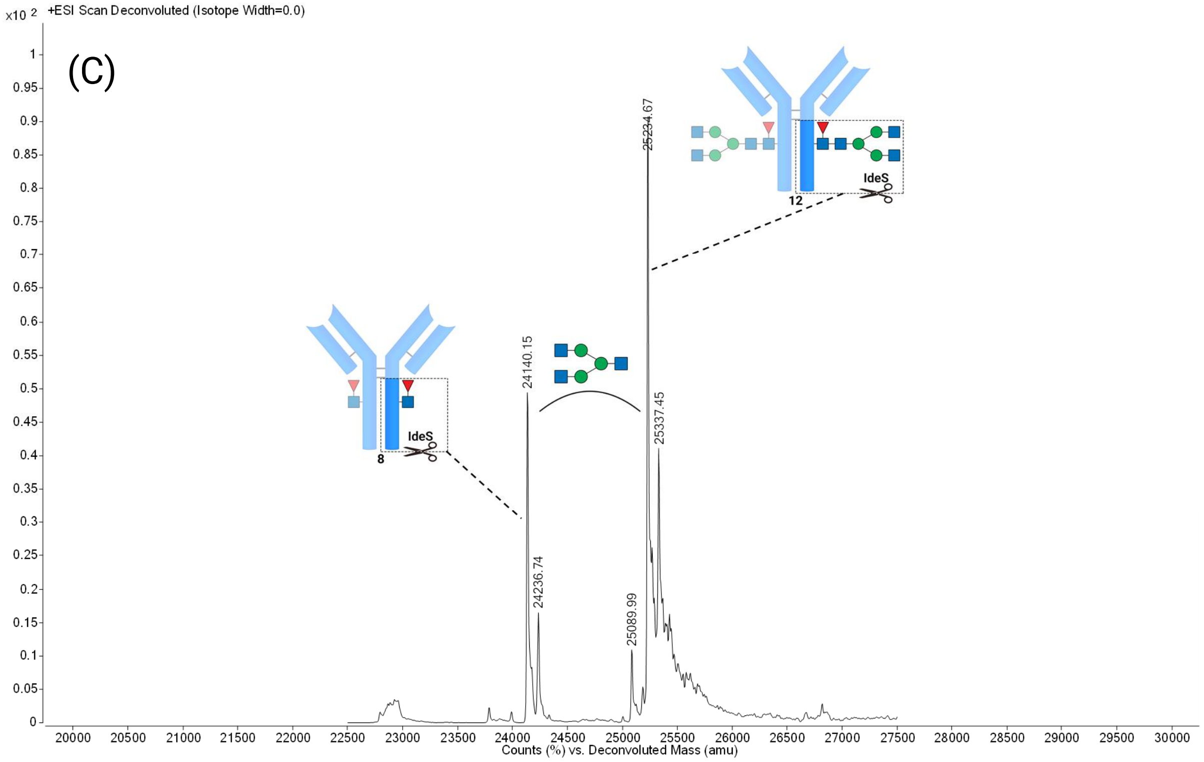

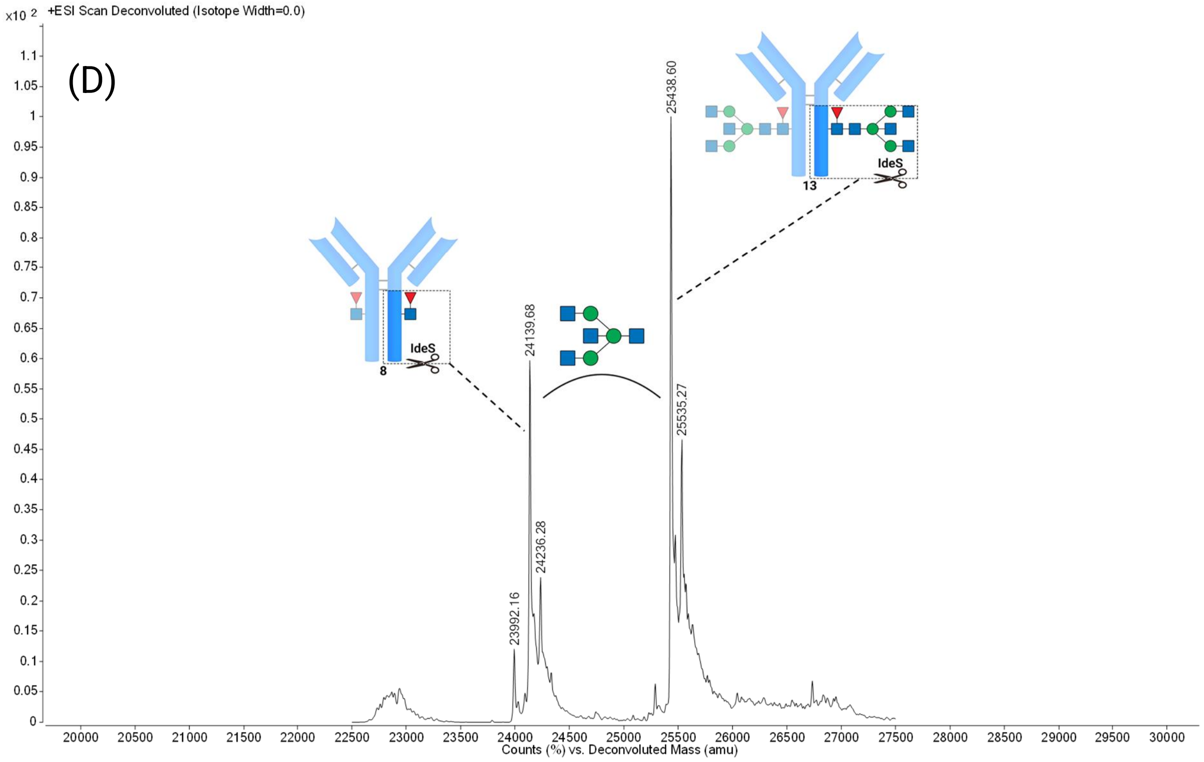

Figure S6. Transglycosylation efficiency. Despite optimized conditions the efficiency of the glycan transfer was not quantitative in a single attempt. Therefore, the semi-finished compounds were purified and resubmitted to the transglycosylation reaction affording the fully glycosylated products as shown in Figure S7-S13. Here, 3-hour incubation and product formation of (A) mAb 10, (B) mAb 11, (C) mAb 12 and (D) mAb 13 is shown. Longer incubation led to hydrolysis of the glycans of the products.

Figure S7. Deconvoluted MS spectrum mAb 7

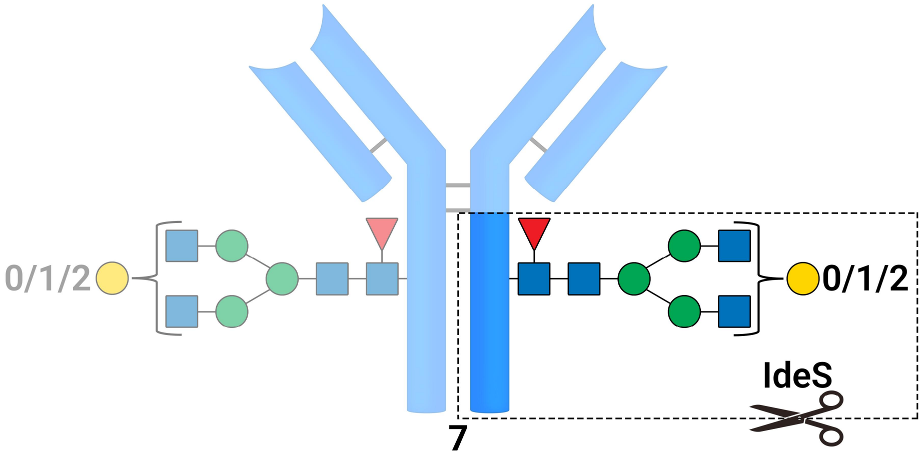

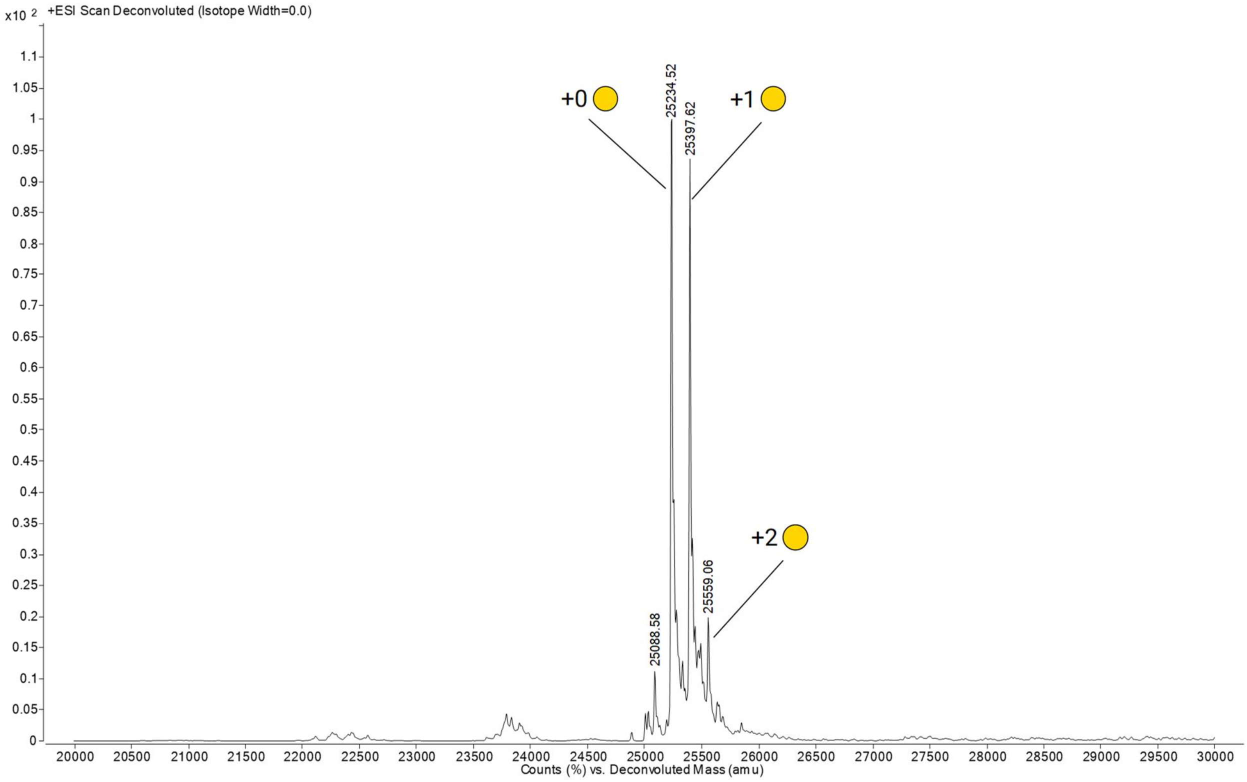

Figure S8. Deconvoluted MS spectrum mAb 8

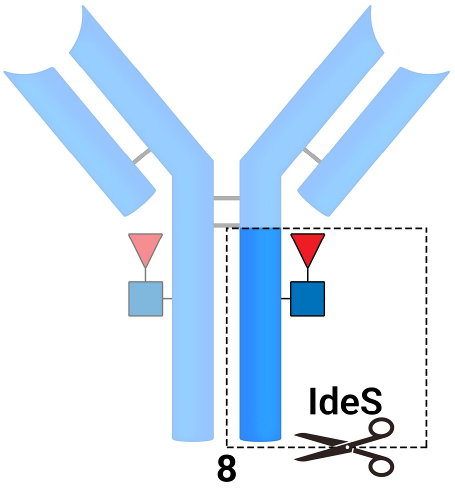

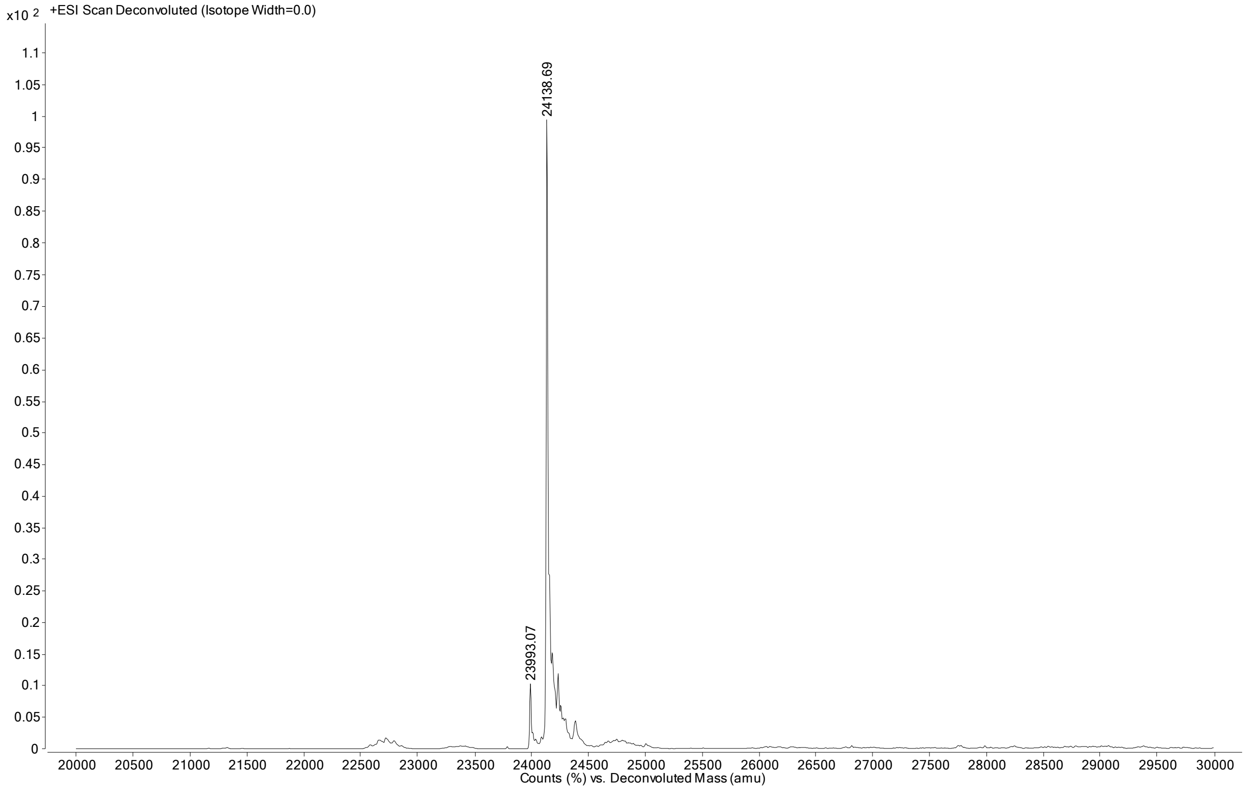

Note: 5-10% is afuco-GlcNAc-trastuzumab (23993.07 Da)

Figure S9. Deconvoluted MS spectrum mAb 9

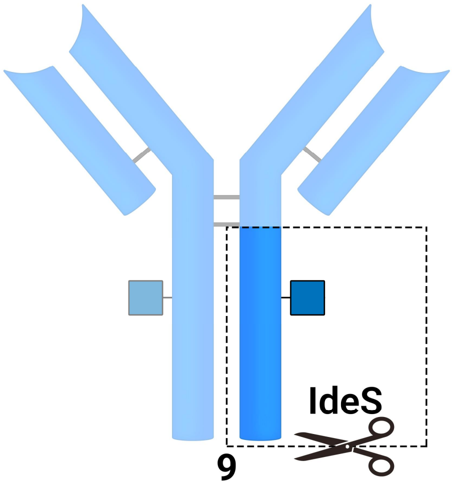

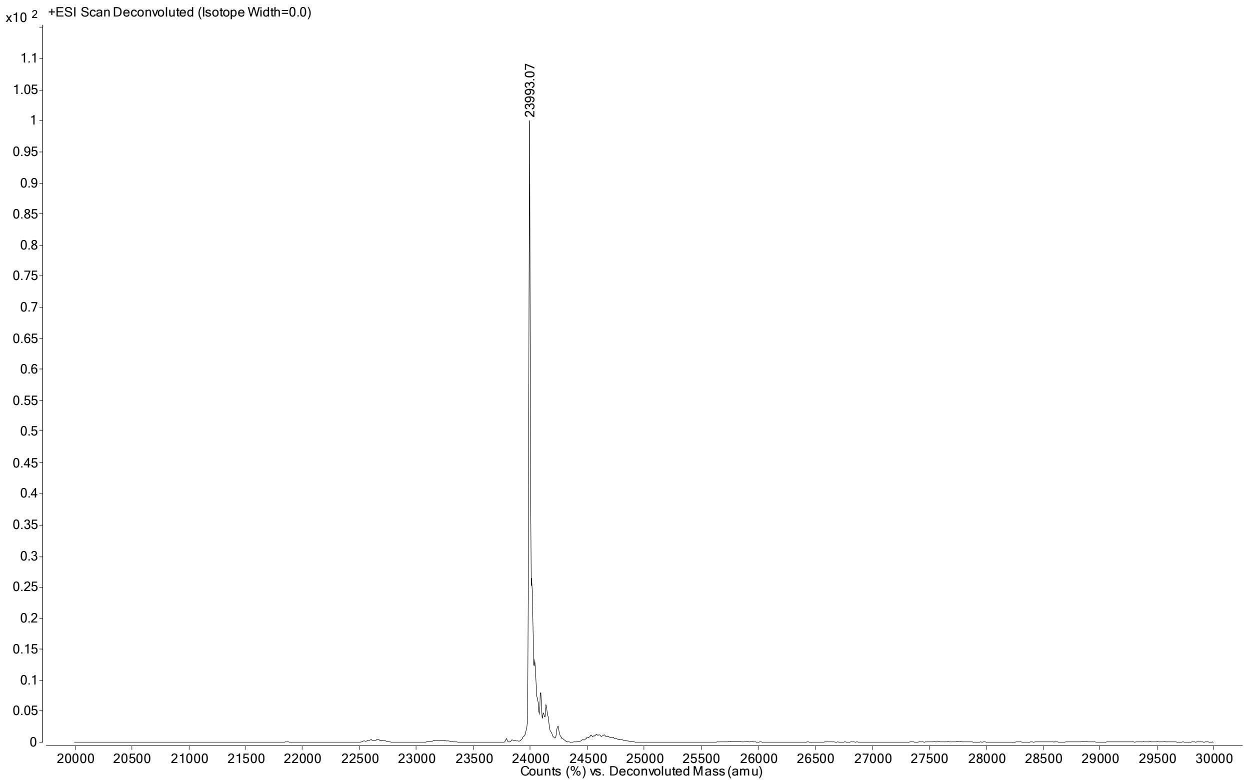

Figure S10. Deconvoluted MS spectrum mAb 10

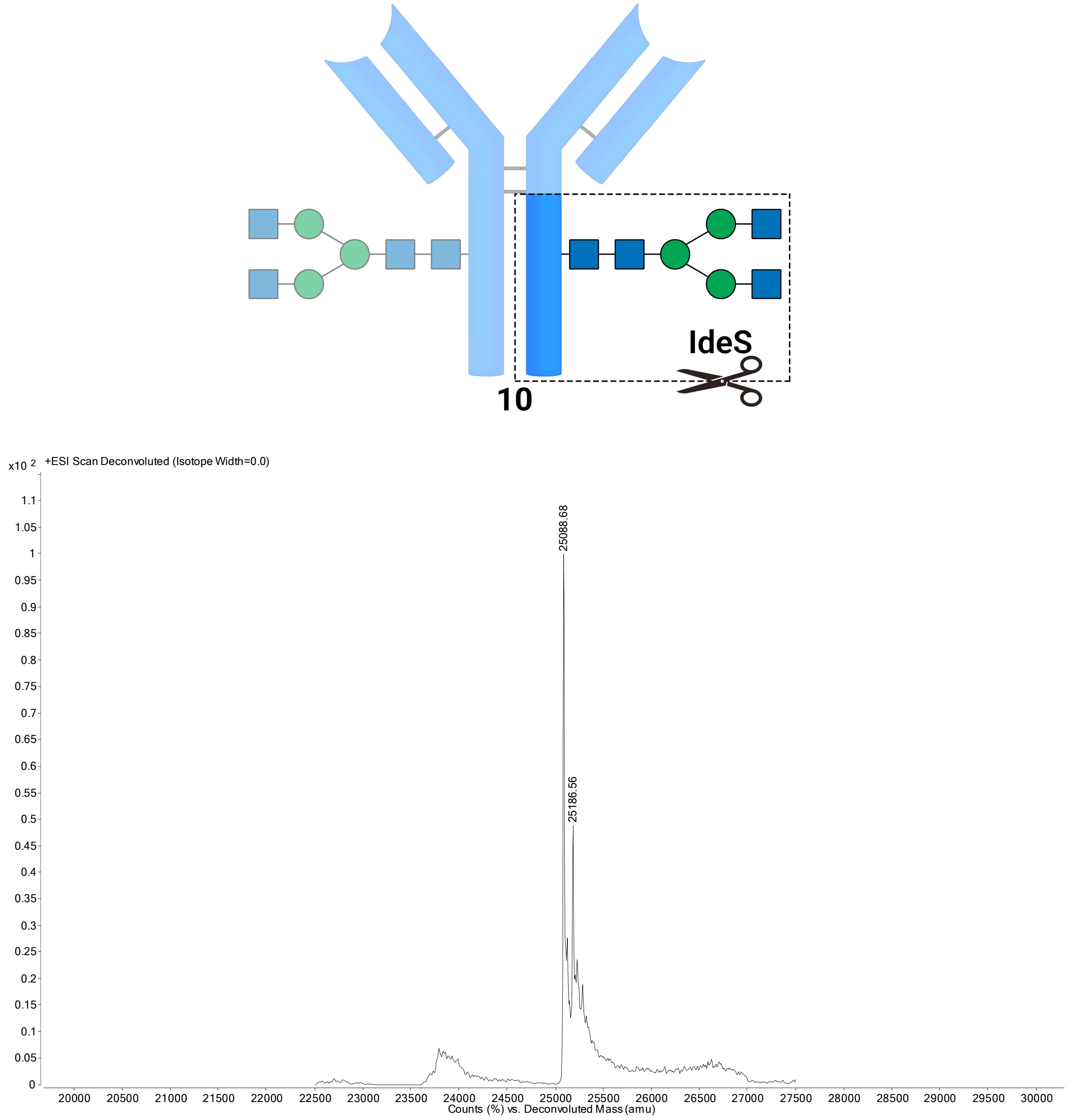

Figure S11. Deconvoluted MS spectrum mAb 11

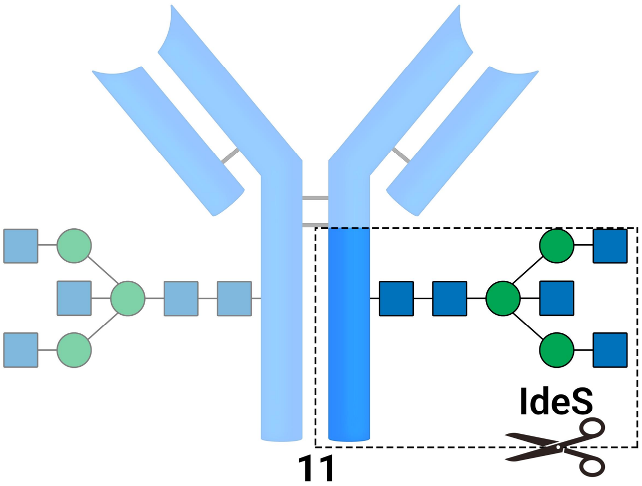

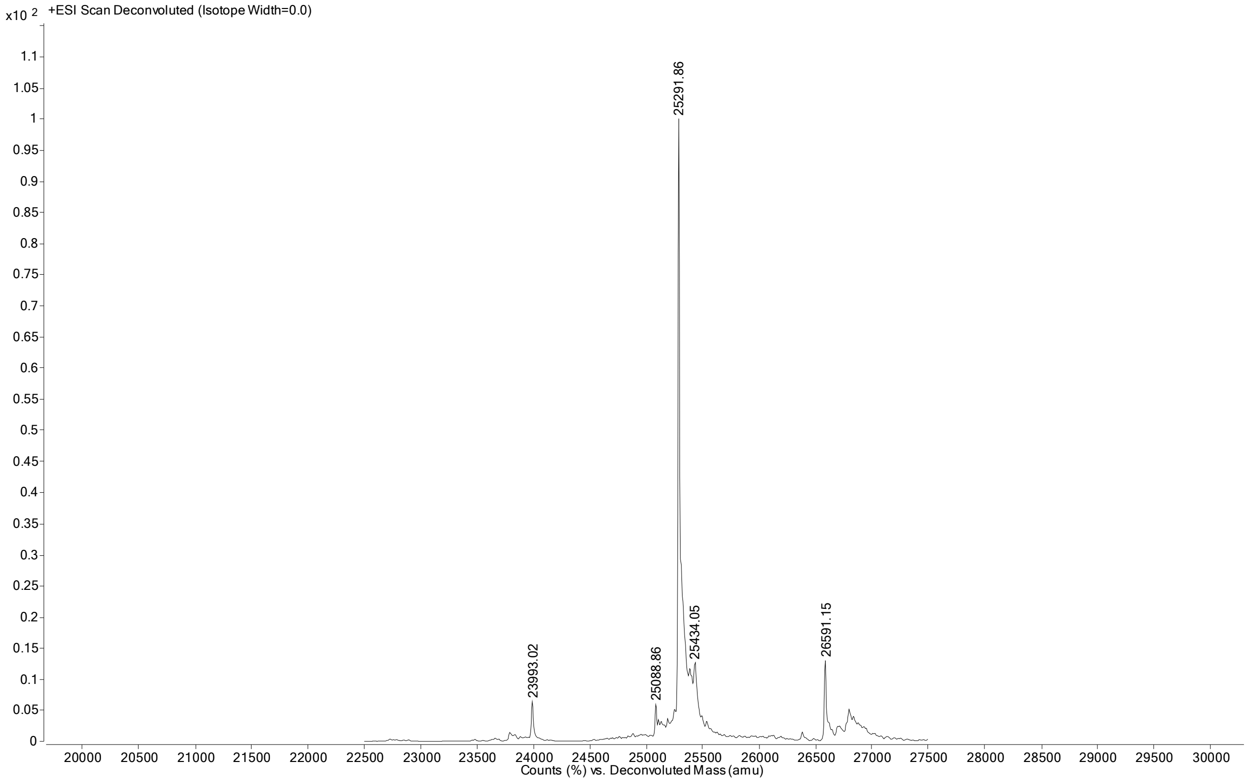

Figure S12. Deconvoluted MS spectrum mAb 12

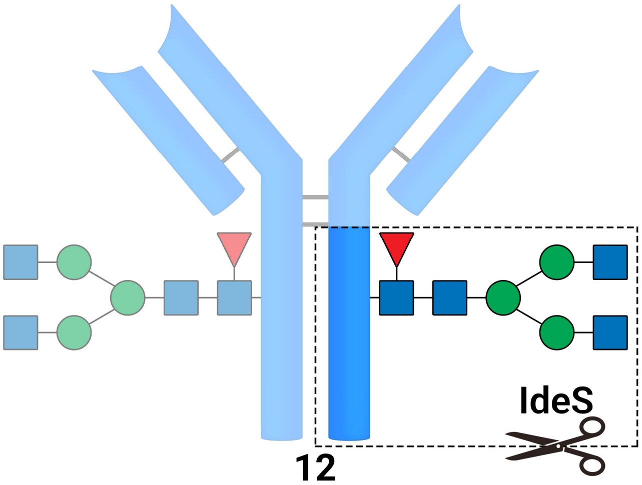

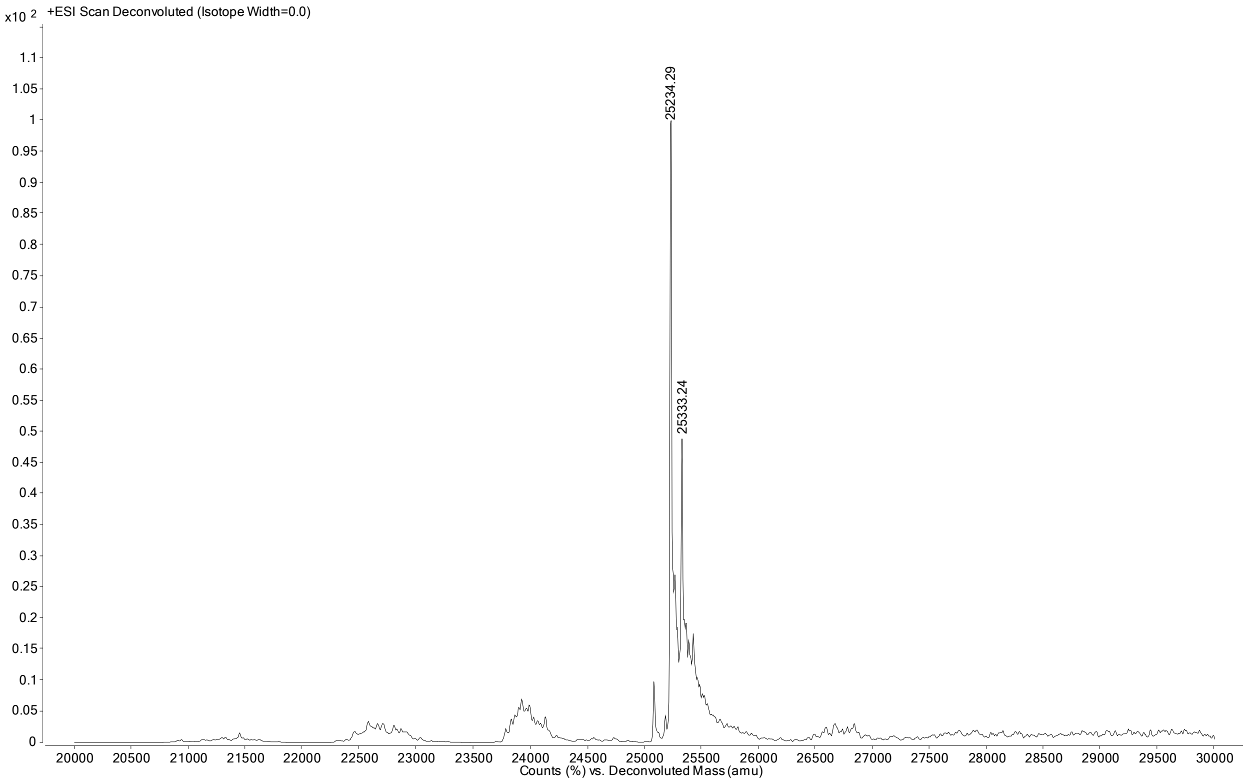

Figure S13. Deconvoluted MS spectrum mAb 13

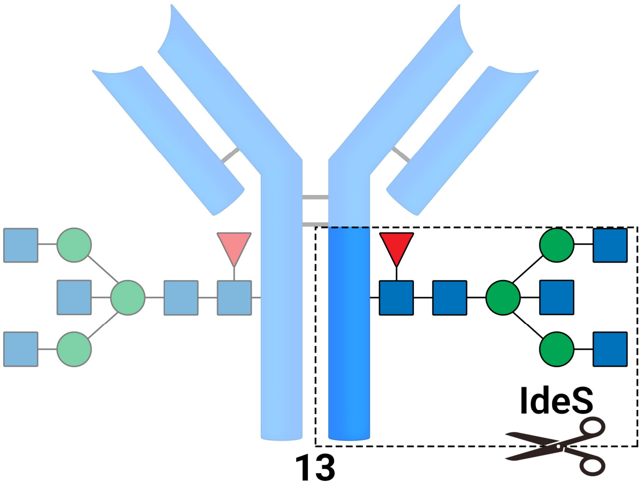

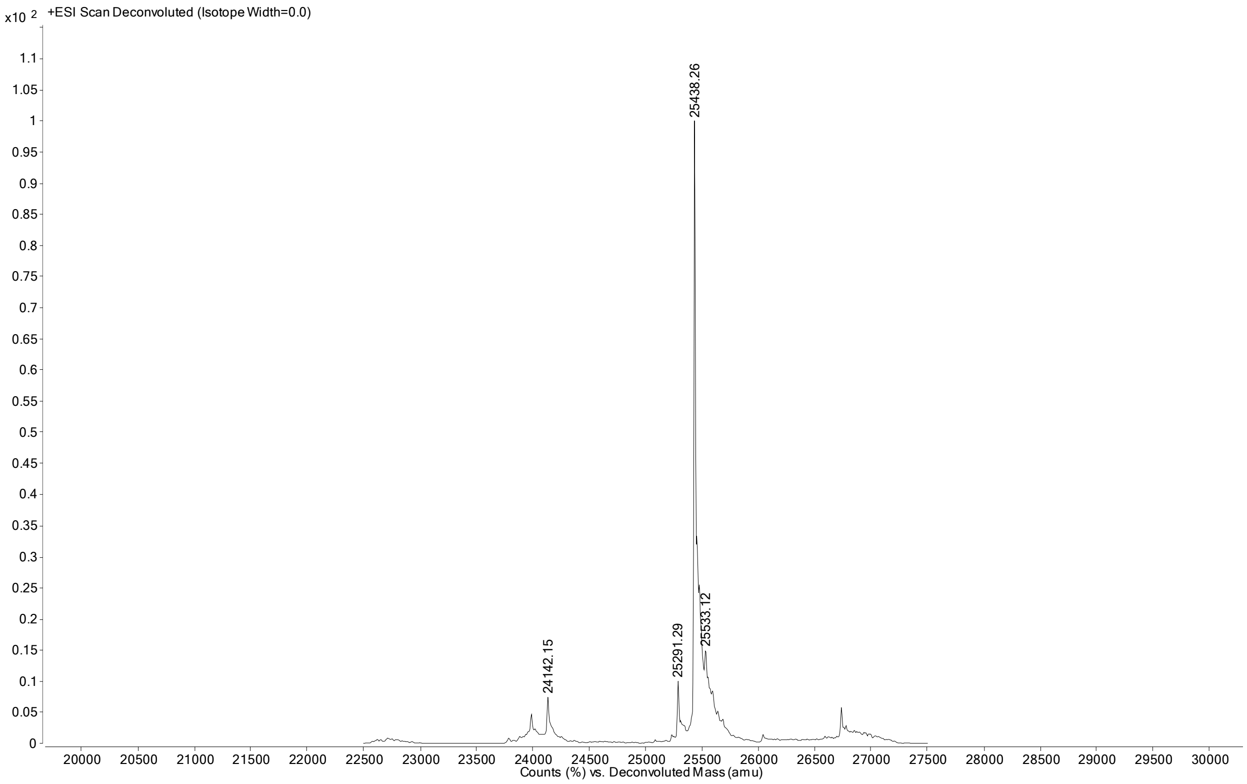

Figure S14. SDS-PAGE gel - Coomassie stain of mAb 7, 8, 10-13

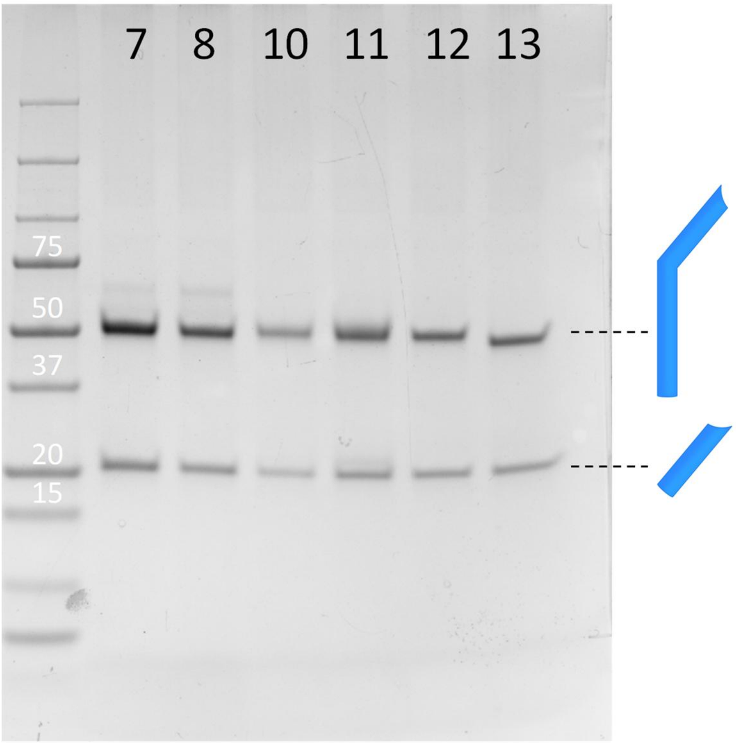

Figure S14. Trastuzumab variants 7, 8, 10-13 run on 4–20% Mini-PROTEAN® TGX™ Precast Protein Gel, 12-well under reducing conditions (in Laemmli sample buffer + 5% β-mercaptoethanol). Ladder: Precision Plus Protein™ All Blue Prestained Protein Standard #1610373. Stain: Coomassie (GelCode™ Blue Stain Reagent).
